# RNA methylomes reveal the m^6^A-mediated regulation of DNA demethylase gene *SlDML2* in tomato fruit ripening

**DOI:** 10.1186/s13059-019-1771-7

**Published:** 2019-08-06

**Authors:** Leilei Zhou, Shiping Tian, Guozheng Qin

**Affiliations:** 1 Key Laboratory of Plant Resources, Institute of Botany, Innovation Academy for Seed Design, Chinese Academy of Sciences, No.20 Nanxincun, Xiangshan, Haidian District, Beijing, 100093 China; 2 University of Chinese Academy of Sciences, Beijing, 100049 China

**Keywords:** Fruit ripening, DNA methylation, mRNA m^6^A methylation, m^6^A RNA methylome, RNA demethylase SlALKBH2, DNA demethylase SlDML2, *Colorless non-ripening*, Tomato

## Abstract

**Background:**

Methylation of nucleotides, notably in the forms of 5-methylcytosine (5mC) in DNA and N^6^-methyladenosine (m^6^A) in mRNA, carries important information for gene regulation. 5mC has been elucidated to participate in the regulation of fruit ripening, whereas the function of m^6^A in this process and the interplay between 5mC and m^6^A remain uncharacterized.

**Results:**

Here, we show that mRNA m^6^A methylation exhibits dynamic changes similar to DNA methylation during tomato fruit ripening. RNA methylome analysis reveals that m^6^A methylation is a prevalent modification in the mRNA of tomato fruit, and the m^6^A sites are enriched around the stop codons and within the 3′ untranslated regions. In the fruit of the ripening-deficient epimutant *Colorless non-ripening* (*Cnr*) which harbors DNA hypermethylation, over 1100 transcripts display increased m^6^A levels, while only 134 transcripts show decreased m^6^A enrichment, suggesting a global increase in m^6^A. The m^6^A deposition is generally negatively correlated with transcript abundance. Further analysis demonstrates that the overall increase in m^6^A methylation in *Cnr* mutant fruit is associated with the decreased expression of RNA demethylase gene *SlALKBH2*, which is regulated by DNA methylation. Interestingly, SlALKBH2 has the ability to bind the transcript of *SlDML2*, a DNA demethylase gene required for tomato fruit ripening, and modulates its stability via m^6^A demethylation. Mutation of *SlALKBH2* decreases the abundance of *SlDML2* mRNA and delays fruit ripening.

**Conclusions:**

Our study identifies a novel layer of gene regulation for key ripening genes and establishes an essential molecular link between DNA methylation and mRNA m^6^A methylation during fruit ripening.

**Electronic supplementary material:**

The online version of this article (10.1186/s13059-019-1771-7) contains supplementary material, which is available to authorized users.

## Background

N^6^-methyladenosine (m^6^A) is considered as the most prevalent internal messenger RNA (mRNA) modification found in eukaryotes, including mammals, plants, flies, and yeasts [1–6]. The m^6^A modification plays multiple functions in mRNA metabolism, including mRNA stability, splicing, translation efficiency, and nuclear export [7–15]. Accumulating evidence suggests that m^6^A affects different developmental and biological processes, such as cancer stem cell proliferation, embryonic and post-embryonic development, cell circadian rhythms, and cell fate decision [16–20], highlighting the biological importance of m^6^A modification. As a dynamic and reversible post-transcriptional modification, the m^6^A methylation in mammals is installed by the methyltransferase complex containing methyltransferase like 3 (METTL3), METTL14, and Wilms’ tumor 1-associating protein (WTAP) [21–24], whereas its removal is mediated by the demethylases fat mass and obesity-associated protein (FTO) and alkylated DNA repair protein AlkB homolog 5 (ALKBH5) [25, 26]. Recognition of the m^6^A-modified transcripts is achieved by the “reader” proteins (such as YTH domain family proteins), which mediate the downstream effects of the m^6^A modification [10, 12, 27]. In plants, the m^6^A methylation machineries were recently characterized in *Arabidopsis thaliana*, the model plant, to regulate shoot stem cell fate, floral transition, and trichome branching [6, 28–32]. However, the relevant knowledge regarding the regulatory mechanisms of m^6^A remains largely unknown. Moreover, the characteristics and functions of m^6^A in physiological processes of horticultural crops such as ripening of a fleshy fruit have not been defined.

Fleshy fruits are important components of human diets, providing essential vitamins and a wide range of “bioactive” compounds that are important for human health, such as carotenoids, polyphenols, plant sterols, and polyunsaturated fatty acids [33]. The ripening of fleshy fruit is an economically important developmental process that impacts fruit nutritional quality and shelf life. Various environmental and internal cues, including light, phytohormones, and developmental genes, participate in the regulation of fruit ripening [33, 34]. More recently, it has been revealed that fruit ripening involves epigenetic regulation, and the transcription of numerous fruit-ripening genes is associated with the DNA methylation status [35–39]. Mutation of *SlDML2*, which encodes a DNA demethylase in tomato, causes genome-wide DNA hypermethylation and dramatic inhibition of fruit ripening [38]. DNA methylation, in the forms of 5-methylcytosine (5mC), is a conserved epigenetic modification that plays broad and critical roles in fundamental biological processes [40–42]. DNA methylation changes the environment of chromatin regions where transcription factors and basic transcription machinery bind, thereby affecting gene expression positively or negatively [40]. Gene-associated DNA methylation can occur in the promoter, which usually represses gene transcription, or within the gene body regions, which is generally associated with high expression levels [42]. In addition to transcription regulation, DNA methylation has been found to modulate mRNA alternative splicing, which occurs at post-transcriptional levels in higher eukaryotes [43, 44]. However, whether DNA methylation influences m^6^A methylation in the process of fruit ripening remains elusive.

In the present study, we show that the overall m^6^A mRNA methylation declines during the ripening of a tomato fruit, which undergoes genome-wide loss of DNA methylation. By contrast, the fruit of the ripening-deficient epimutant *Colorless non-ripening* (*Cnr*), which shows genome-wide DNA hypermethylation [36], exhibits higher m^6^A level compared with the fruit of the wild type. The *Cnr* mutant has been previously characterized, using positional cloning, to harbor a naturally occurring epigenetic mutation in a gene encoding an SBP-box transcription factor [35]. Transcriptome-wide characterization of m^6^A methylation profiles demonstrates that m^6^A represents a prevalent modification in mRNA of tomato fruit, and the abundance of m^6^A in transcripts of a large number of genes alters substantially during fruit ripening or in the fruit of the *Cnr* mutant. We further demonstrate that DNA methylation regulates the transcription of *SlALKBH2* that encodes an m^6^A demethylase located in the endoplasmic reticulum. Strikingly, SlALKBH2 has the ability to bind *SlDML2* mRNA and mediate its m^6^A demethylation, thus modulating *SlDML2* mRNA stability. Mutation of *SlALKBH2* by CRISPR/Cas9 gene-editing system decreases *SlDML2* mRNA level and delays fruit ripening. Our findings reveal that DNA methylation affects mRNA m^6^A methylation by targeting *SlALKBH2*, which in turn acts on *SlDML2* by a feedback loop to regulate fruit ripening.

## Results

### mRNA m^6^A methylation exhibits dynamic changes similar to DNA methylation during tomato fruit ripening

**Fig. 1:**
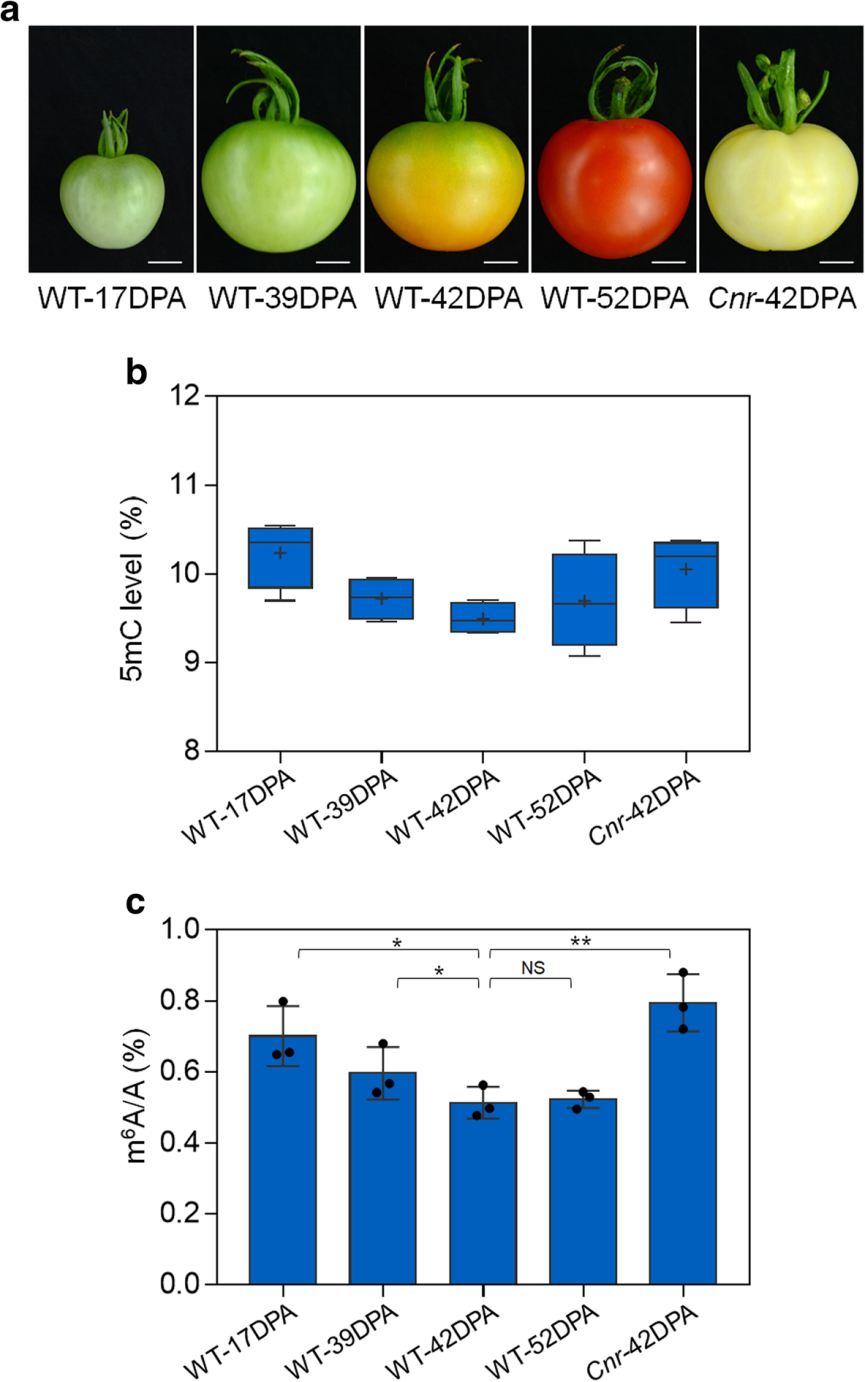
Dynamics of DNA methylation (5mC) and mRNA m^6^A methylation in tomato fruit ripening. **a** Images of wild-type (WT) fruit at different ripening stages and *Cnr* fruit at 42 DPA. DPA, days post-anthesis; scale bar = 1 cm. **b** Relative 5mC levels of WT and *Cnr* fruit shown in **a**. For 5mC assay, 100 ng of genomic DNA was detected in each sample by MethylFlash™ methylated DNA quantification kit. 5mC level in each sample was normalized to that of the positive control according to the manufacturer’s instructions. The plus sign represents the average in each box. **c** LC-MS/MS assay showing the amount of mRNA m^6^A in WT and *Cnr* fruit shown in **a**. Data are presented as mean ± standard deviation (*n* = 3). Asterisks indicate significant differences (*P < 0.05, **P < 0.01; Student’s *t* test). NS, no significance

DNA methylation (5mC) has been proven to play crucial roles in the regulation of tomato fruit ripening [36–38]. We examined the changes in 5mC levels in the progress of tomato fruit ripening (Fig. 1a) and found that, consistent with previous reports [36, 38], the overall 5mC levels declined as fruit ripening (Fig. 1b). The spontaneous epimutant *Cnr*, which displays a colorless non-ripe phenotype (Fig. 1a), exhibits hypermethylation compared with the wild type (Fig. 1b). We then assessed the mRNA m^6^A methylation levels in the same samples by using LC-MS/MS assay (Additional file 1: Figure S1). The results showed that the overall mRNA m^6^A levels decreased during fruit ripening but exhibited an obviously higher level in the DNA hypermethylated *Cnr* mutant (Fig. 1c). These data indicated that DNA 5mC and mRNA m^6^A harbor a similar dynamic change during tomato fruit ripening, as well as in the *Cnr* mutant. We hypothesize that there may exist a correlation between these two nucleic acid modifications and mRNA m^6^A may participate in regulating tomato fruit ripening as DNA methylation.

### m^6^A methylation is a common feature of mRNAs in tomato fruit as revealed by m^6^A methylome

**Fig. 2:**
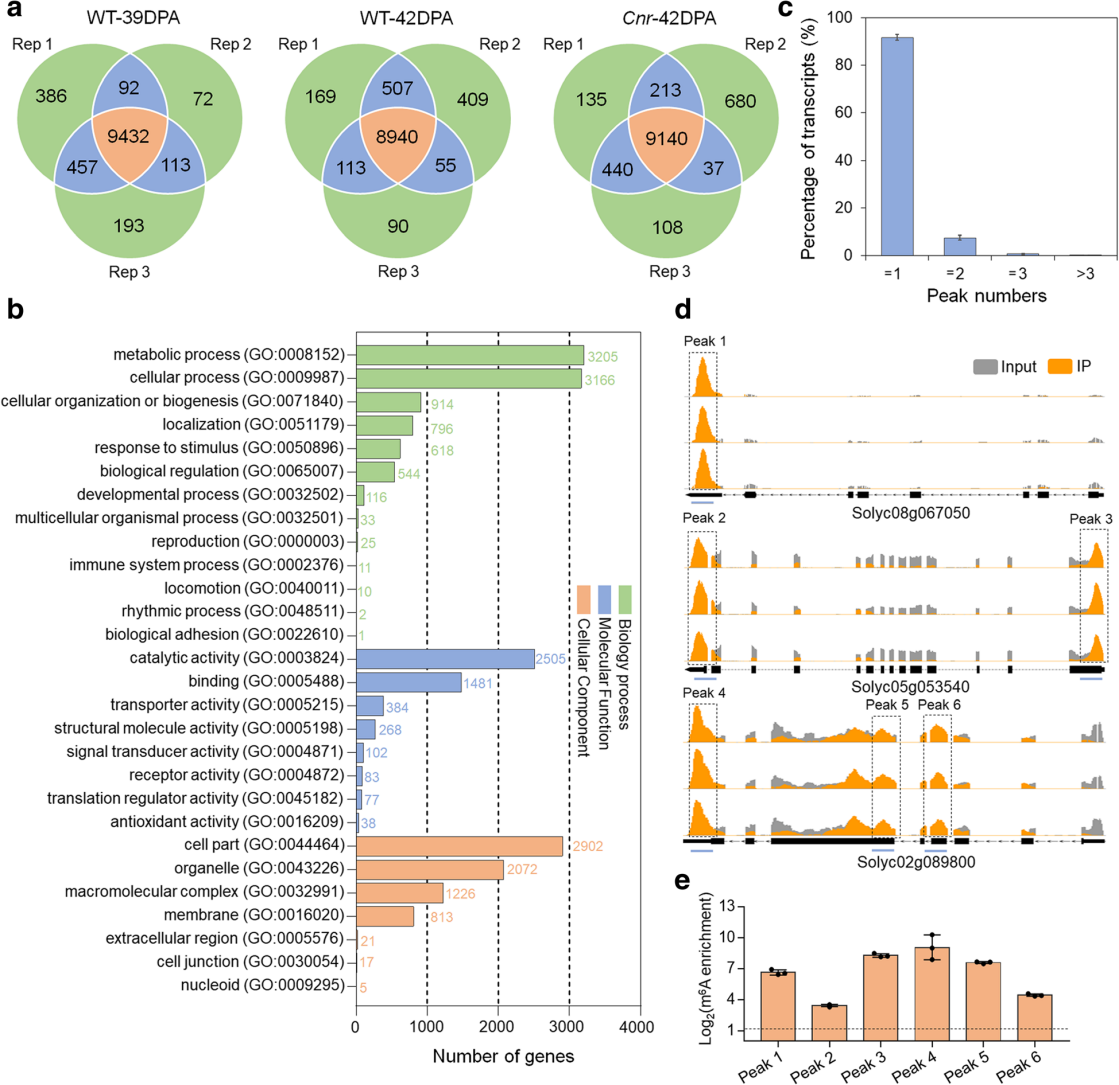
Transcriptome-wide m^6^A methylation profiles in tomato fruit. **a** Venn diagrams showing the overlap of m^6^A peaks identified in three independent m^6^A-seq experiments on wild-type (WT) fruit at 39 DPA and 42 DPA and *Cnr* fruit at 42 DPA. Rep, replicate; DPA, days post-anthesis. Only the peaks identified in all three biological replicates were regarded as confident peaks and used for subsequent data analysis. **b** Gene Ontology (GO) analysis of the biological process, molecular function, and cellular component for the m^6^A-containing transcripts identified in m^6^A-seq. **c** Proportions of the m^6^A-modified transcripts containing different m^6^A peak numbers. Error bars represent the standard deviation of three different m^6^A-seq experiments. **d** Examples of m^6^A-modified transcripts containing one m^6^A peak, two m^6^A peaks, and three m^6^A peaks in WT fruit at 42 DPA. The black dot line rectangles indicate the positions of m^6^A peaks, which are named as peaks 1–6. The blue lines indicate the positions of amplification fragments in the following m^6^A-immunoprecipitation (IP)-qPCR. **e** Validations of the m^6^A peaks shown in **d** by m^6^A-IP-qPCR. Data are presented as mean ± standard deviation (*n* = 3)

To investigate whether a correlation exists between DNA methylation and mRNA m^6^A methylation, and whether m^6^A modification is involved in the regulation of tomato fruit ripening, we performed m^6^A-seq [45] to profile transcriptome-wide m^6^A methylation (m^6^A methylome) on the fruit of wild type at 39 days post-anthesis (DPA; the “mature green” ripening stage) and 42 DPA (the “breaker” ripening stage), in addition to the fruit of the DNA hypermethylated mutant *Cnr* at 42 DPA. The mRNAs from different samples were fragmented into ~ 100 nucleotide-long oligonucleotides (input) prior to immunoprecipitation using an anti-m^6^A affinity purified antibody. Libraries were prepared from input control as well as immunoprecipitated fragments and subjected to massively parallel sequencing. We performed three replicate m^6^A-seq experiments, in which the mRNA samples were independently prepared. High Pearson correlation coefficient was found between biological replicates, representing highly reproducible (Additional file 1: Figure S2). A total of 20–30 million reads were generated for each library, and there were 19–28 million distinct reads uniquely aligned to the tomato genome SL3.0 (~ 95% mapping to unique loci) (Additional file 2: Table S1). A peak detection algorithm was used to identify m^6^A peaks with an estimated false discovery rate (FDR) < 0.05 [45]. Only m^6^A peaks consistently detected in all three biological replicates for each sample, which we called high-confidence m^6^A peaks, were used for subsequent analysis. We identified 9432 and 8940 high-confidence m^6^A peaks within 9436 and 9023 gene transcripts, in the wild-type fruit at 39 DPA and 42 DPA, respectively, and 9140 m^6^A peaks within 9442 gene transcripts in the *Cnr* mutant at 42 DPA (Fig. 2a; Additional file 3: Table S2-S4). Gene Ontology (GO) enrichment analysis of m^6^A-containing transcripts revealed a potential function of m^6^A modification in multiple signaling pathways and cellular processes (Fig. 2b).

Based on these results, we estimated that the transcriptome of tomato fruit contains 0.5–0.6 m^6^A peaks per actively expressed transcript (Additional file 4: Table S5). These levels are comparable with those obtained in *Arabidopsis* or mammals [2, 7, 46]. Of the gene transcripts containing m^6^A modification, most (91.73%) contain one m^6^A peak, while 7.47% exhibit two m^6^A peaks, 0.69% exhibit three peaks, and 0.11% exhibit more than three peaks (Fig. 2c).

We then validate the m^6^A-seq results with independent m^6^A-immunoprecipitation (IP)-qPCR. Using this method, we verified the presence of m^6^A within *arginine N-methyltransferase* (Solyc08g067050), *dihydroxy-acid dehydratase* (Solyc05g053540), and *nuclear matrix constituent protein 1-like* (Solyc02g089800) (Fig. 2d). These mRNAs were chosen for the validation of m^6^A presence in transcripts with a single methylation peak (Solyc08g067050) as well as those with multiple m^6^A peaks (Solyc05g053540 and Solyc02g089800). As expected, we observed substantial enrichment of these genes after mRNA immunoprecipitation with the m^6^A-specific antibody compared with the input control (Fig. 2e). These results indicated that our m^6^A-seq data were accurate and robust.

Collectively, these data demonstrated that m^6^A, which appears in a substantial fraction of the transcriptome, is a common feature of mRNA in tomato fruit, and m^6^A-containing transcripts are related to a variety of biological pathways.

### m^6^A distribution and sequence motif in tomato fruit

**Fig. 3:**
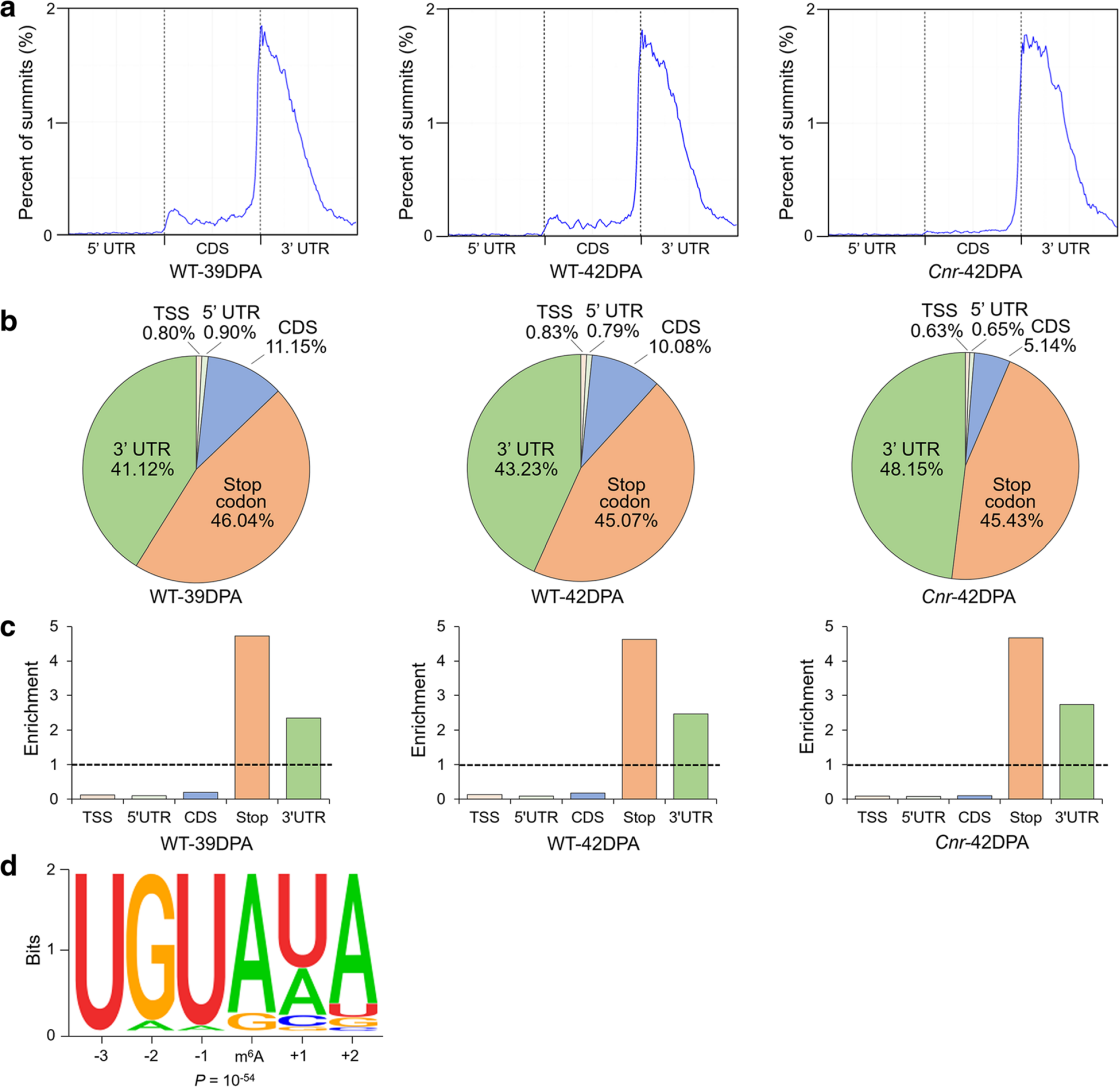
Characteristics of m^6^A localization and sequence motif in tomato fruit. **a** Metagenomic profiles of peak summit distributions along the transcripts composed of three rescaled non-overlapping segments (5′ UTR, CDS, and 3′ UTR). UTR, untranslated region; CDS, coding sequence. **b** Pie charts depicting the fraction of m^6^A peak summits in five non-overlapping transcript segments. TSS, transcription start site. **c** Relative enrichment of m^6^A peak summits in five non-overlapping transcript segments. **a**–**c** The results for wild-type (WT) fruit at 39 DPA and 42 DPA and *Cnr* fruit at 42 DPA. DPA, days post-anthesis. **d** Sequence motif identified within m^6^A peaks by HOMER (http://homer.ucsd.edu/homer/)

We next characterized the distribution of m^6^A peaks in the whole transcriptome of tomato fruit. The metagenomic profiles of m^6^A peaks in all three samples (wild-type fruit at 39 DPA and 42 DPA and *Cnr* epimutant at 42 DPA) indicated that m^6^A modifications were highly enriched around the stop codon and within the 3′ untranslated region (UTR) (Fig. 3a), consistent with the m^6^A distribution in *Arabidopsis* [31]. To confirm the distribution of m^6^A within the transcript, we divided the transcript into five non-overlapping segments: transcription start site (TSS), 5′ UTR, coding sequence (CDS), stop codon, and 3′ UTR. Each m^6^A peak was assigned to one of five transcript segments. The stop codon segment (100-nucleotide window centered on the stop codon) appeared to be greatly enriched in m^6^A peaks, and 45.07 to 46.04% of the peaks from different samples fell into this segment (Fig. 3b). The enrichment of m^6^A peaks in the 3′ UTR was also revealed, which was comparable to that in the stop codon (Fig. 3b). After segment normalization by the relative fraction that each segment occupied in the transcriptome, we observed that m^6^A is exclusively enriched around the stop codon and within the 3′ UTR, with stop codon peaks being more pronounced than 3′ UTR peaks (Fig. 3c). Overall, the distribution of m^6^A peaks did not display dramatic changes between the samples.

To identify the sequence motifs that are enriched within the m^6^A peaks in tomato fruit, hypergeometric optimization of motif enrichment (HOMER; http://homer.ucsd.edu/homer/) was applied [47]. Clustering of m^6^A peaks using HOMER did not identify previously established RRACH consensus sequence observed in mammals and yeasts [1, 7, 48], where R represents adenosine (A) or guanosine (G), underlined A indicates m^6^A, and H represents A, cytidine (C), or uridine (U), in our data set, but we did identify a UGUAYY sequence motif that was previously observed in *Arabidopsis* [31], where Y represents A, G, U, or C (Fig. 3d). This demonstrated that the sequence motif for m^6^A methylation is conserved among *Arabidopsis* and tomato.

### DNA hypermethylated mutant *Cnr* shows overall increase in m^6^A mRNA methylation

**Fig. 4:**
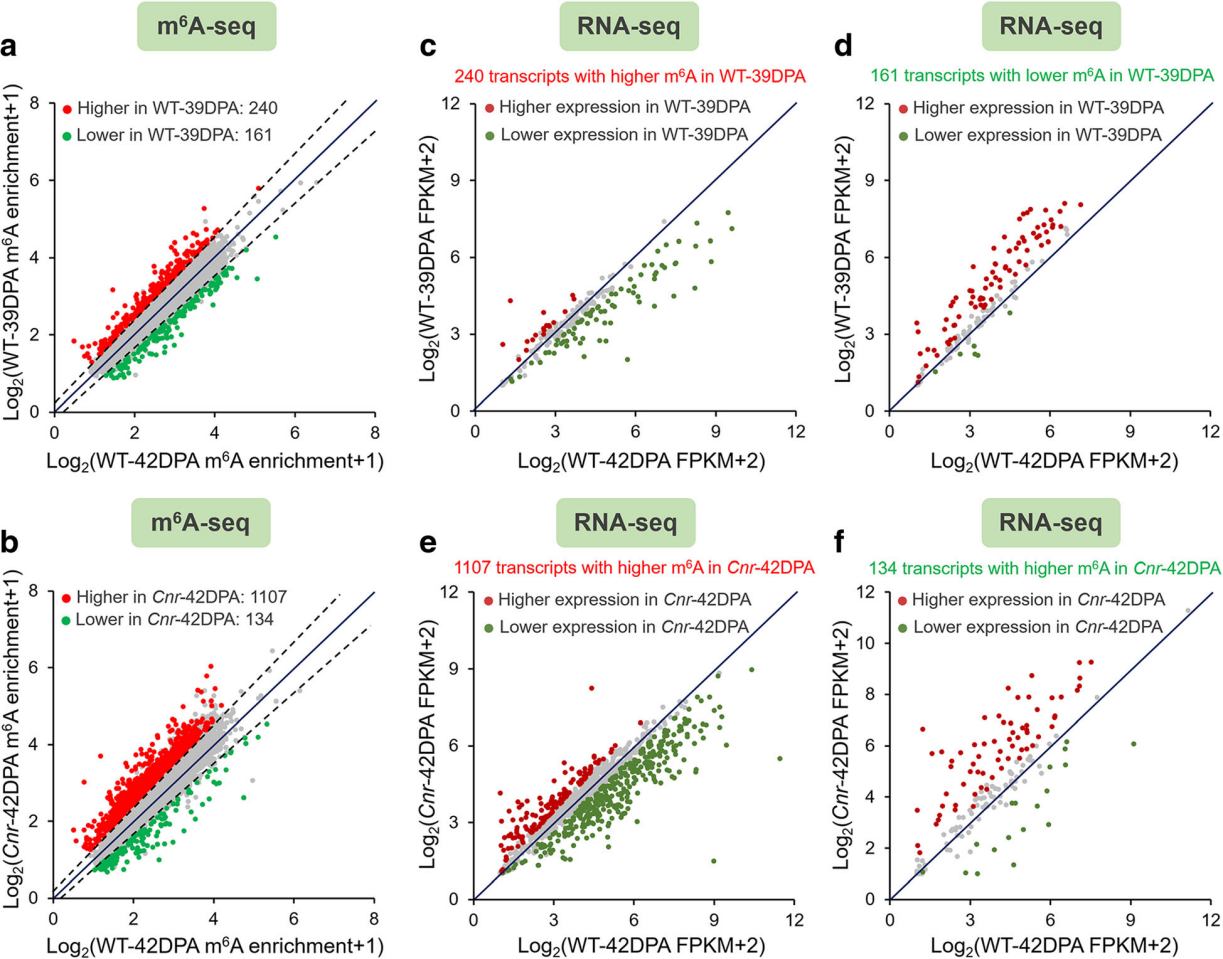
m^6^A methylation is generally negatively correlated with transcript abundance. **a** Scatter plots showing transcripts with differential m^6^A enrichment between wild-type (WT) fruit at 39 DPA and 42 DPA. The m^6^A-modified mRNAs with significantly higher and lower peak enrichment in 39 DPA WT fruit compared to 42 DPA WT fruit are highlighted in red and green, respectively (fold change ≥ 1.5; *P* value < 0.05). **b** Scatter plots showing the transcripts with differential m^6^A enrichment between WT fruit at 42 DPA and *Cnr* fruit at 42 DPA. The m^6^A-modified mRNAs with significantly higher and lower peak enrichment in *Cnr* fruit compared to WT fruit are highlighted in red and green, respectively (fold change ≥ 1.5; *P* value < 0.05). **c** Expression of m^6^A-modified transcripts that exhibit significantly higher peak enrichment in 39 DPA WT fruit compared to 42 DPA WT fruit. **d** Expression of m^6^A-modified transcripts that exhibit significantly lower peak enrichment in 39 DPA WT fruit compared to 42 DPA WT fruit. **c**, **d** Transcripts with significantly higher and lower levels in 39 DPA WT fruit compared to 42 DPA WT fruit are highlighted in red and green, respectively (fold change ≥ 1.5; *P* value < 0.05). **e** Expression of m^6^A-modified transcripts that exhibit significantly higher peak enrichment in *Cnr* fruit compared to WT fruit. **f** Expression of m^6^A-modified transcripts that exhibit significantly lower peak enrichment in *Cnr* fruit compared to WT fruit. **e**, **f** Transcripts with significantly higher and lower levels in *Cnr* fruit compared to WT fruit are highlighted in red and green, respectively (fold change ≥ 1.5; *P* value < 0.05). Gene expression analysis was performed by RNA-seq. FPKM, fragments per kilobase of exon per million mapped fragments

To gain insight into the functional relationship between DNA methylation and mRNA m^6^A methylation, and the potential roles of m^6^A in the regulation of fruit ripening, we compared m^6^A methylomes between the samples. A total of 401 transcripts with differential m^6^A levels (fold change ≥1.5; *P* value < 0.05) between 39 DPA and 42 DPA wild-type fruit were identified in all three biological replicates, among which 240 transcripts (Additional file 5: Table S6) exhibited higher m^6^A levels and 161 transcripts (Additional file 5: Table S7) displayed lower m^6^A levels in 39 DPA wild-type fruit compared to 42 DPA wild-type fruit (Fig. 4a). By contrast, we identified 1241 transcripts that exhibited differential m^6^A levels (fold change ≥ 1.5; *P* value < 0.05) between 42 DPA *Cnr* mutant fruit and 42 DPA wild-type fruit. A total of 1107 transcripts (Additional file 5: Table S8) displayed higher levels of m^6^A enrichment in the *Cnr* mutant compared to the wild type, whereas only 134 transcripts (Additional file 5: Table S9) showed decreased m^6^A levels (Fig. 4b), suggesting a global increase in m^6^A methylation. This is in accordance with the result of LC-MS/MS assay (Fig. 1c), showing that m^6^A levels increased markedly in the *Cnr* mutant.

m^6^A deposition has been reported to influence mRNA abundance [6, 10, 29, 49]. To evaluate whether there is a potential correlation between m^6^A mRNA methylation and gene transcript levels in tomato fruit, RNA-seq analyses (Fig. 4c–f) were performed with three highly reproducible biological replicates (Additional file 1: Figure S3). Comparison of differentially expressed genes (fold change ≥ 1.5; *P* value < 0.05) (Additional file 6: Table S10-S11) with our list of transcripts showing altered m^6^A levels revealed that, among the 1107 transcripts with higher m^6^A levels in fruit of *Cnr* mutant compared to wild type, only 136 showed higher expression levels, whereas 349 exhibited lower expression levels (Fig. 4e; Additional file 7: Table S12). Accordingly, among the 134 transcripts with lower m^6^A levels in the fruit of *Cnr* mutant compared to wild type, 66 and 18 displayed higher and lower expression levels, respectively (Fig. 4f; Additional file 7: Table S13). These data suggest that m^6^A methylation is generally negatively correlated with the abundance of the transcripts. Similar results were observed in wild-type fruit between 39 DPA and 42 DPA (Fig. 4c, d; Additional file 7: Table S14-S15). Notably, hundreds of ripening-induced and ripening-repressed genes, which exhibit significantly increased or decreased expression in 42 DPA wild-type fruit compared to 39 DPA wild-type fruit, show changed m^6^A levels during fruit ripening (Additional file 8: Table S16) or in the *Cnr* mutant (Additional file 8: Table S17), implicating the involvement of m^6^A modification in the regulation of fruit ripening.

### Transcripts of fruit-ripening genes exhibit increased m^6^A levels in the *Cnr* mutant

**Fig. 5:**
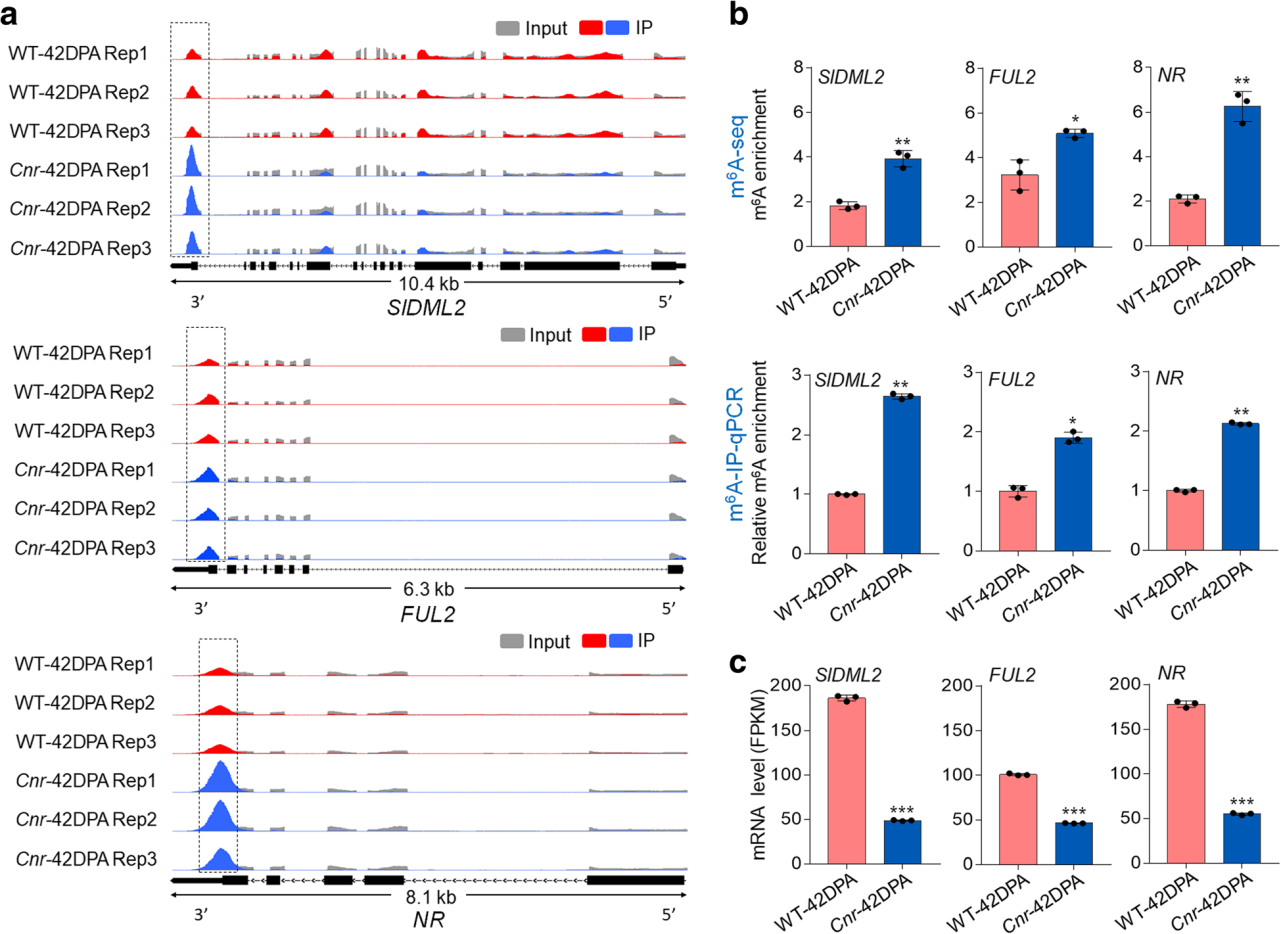
Changes in m^6^A levels in transcripts of specific ripening-related genes in the *Cnr* mutant. **a** Integrated Genome Browser (IGB) tracks displaying m^6^A-seq read distributions in *DEMETER-like DNA demethylase 2* (*SlDML2*), *fruitfull 2* (*FUL2*), and *never-ripe* (*NR*) transcripts. The black dot line rectangles indicate the position of m^6^A peaks with significantly increased m^6^A enrichment (fold changes ≥ 1.5 and *P* value < 0.05) in *Cnr* fruit compared to wild-type (WT) fruit. **b** Validations of the m^6^A enrichment by m^6^A-immunoprecipitation (IP)-qPCR. **c** Gene expression level of *SlDML2*, *FUL2*, and *NR* revealed by RNA-seq. **b**, **c** Error bars represent the standard deviation of three independent experiments. Asterisks indicate significant differences (*P < 0.05, **P < 0.01, ***P < 0.001; Student’s *t* test)

In the m^6^A-seq analysis, we found that transcripts of several well-known fruit-ripening genes, including *DEMETER-like DNA demethylase 2* (*SlDML2*), *fruitfull 2* (*FUL2*), and *never-ripe* (*NR*), exhibit significantly increased m^6^A levels in the *Cnr* mutant (Fig. 5a; Additional file 5: Table S8). *SlDML2* encodes a DNA demethylase [37], while *FUL2* and *NR* encode a MADS-box transcription factor and an ethylene receptor, respectively [50, 51]. The m^6^A peaks were enriched near the stop codon or within 3′ UTR in mRNAs of these genes, and the changes in m^6^A levels were observed in all three biological replications (Fig. 5a), indicating the reproducibility of our m^6^A-seq data. m^6^A-IP-qPCR confirmed the results of m^6^A-seq and demonstrated that the mRNAs of *SlDML2*, *FUL2*, and *NR* displayed higher levels of m^6^A enrichment in the fruit of *Cnr* mutant compared with the wild-type (Fig. 5b). The transcript levels of these three genes decreased significantly in the *Cnr* mutant as revealed by transcriptome analysis (Fig. 5c), implying a negative correlation between m^6^A modification and mRNA abundance. It should be noted that the mRNAs of *SlDML2* and *NR*, but not *FUL2*, exhibited lower levels of m^6^A enrichment, accompanied by higher transcript levels, in the fruit of wild type at 42 DPA compared with the fruit of wild type at 39 DPA (Additional file 1: Figure S4).

### *SlALKBH2* is a putative m^6^A RNA demethylase gene that declines in the *Cnr* mutant

Having observing the changes in m^6^A levels in a large number of transcripts (Fig. 4a, b), including those of well-known fruit-ripening genes (Fig. 5a), in the *Cnr* mutant, we next examined the underlying mechanisms. Since *Cnr* is an epimutant that displays DNA hypermethylation, the variation in m^6^A might result from DNA methylation-mediated expression alteration of m^6^A methylation machinery, i.e., RNA methyltransferases and demethylases [15]. We speculate that the substantial increase in m^6^A levels in the *Cnr* mutant is mainly caused by downregulation of RNA demethylase genes because DNA methylation is usually negatively correlated with target gene expression.

**Fig. 6:**
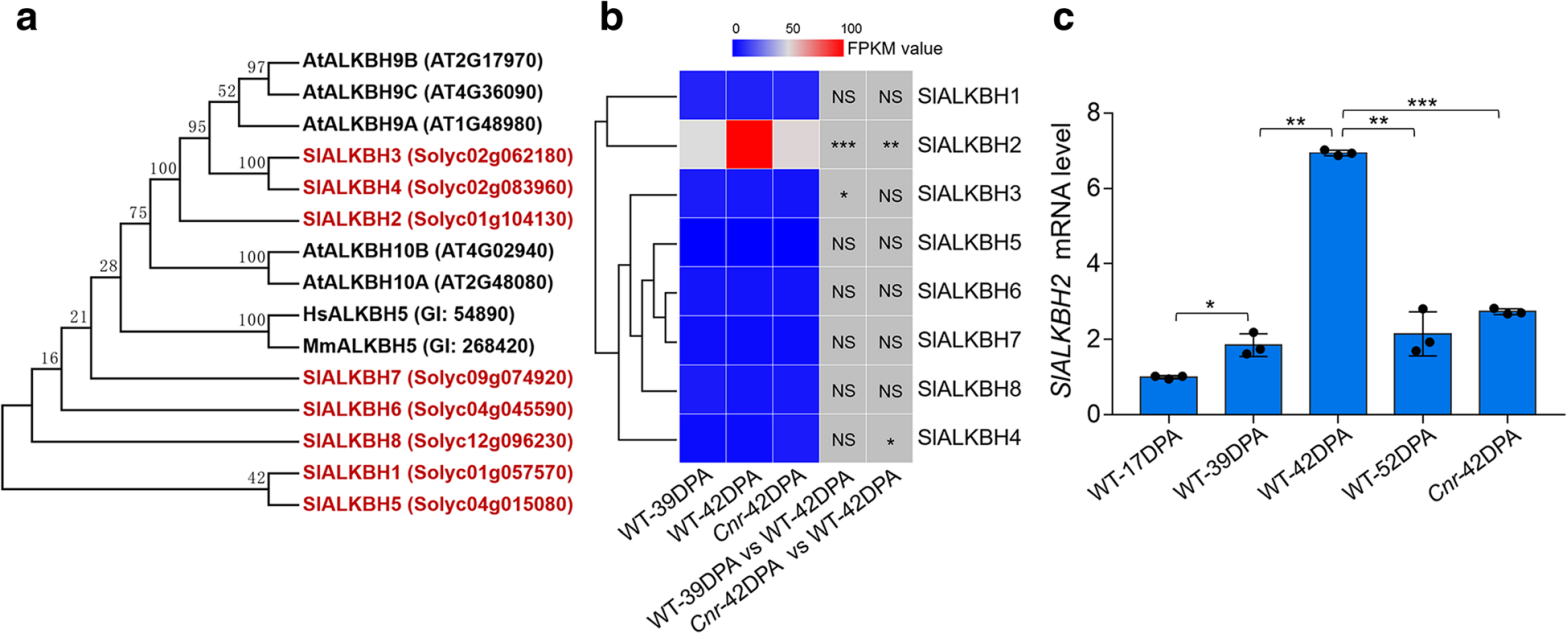
Expression analyses of tomato *ALKBH* genes reveal the involvement of *SlALKBH2* in fruit ripening. **a** Phylogenetic analysis of tomato m^6^A RNA demethylases (ALKBHs). Phylogenetic tree of the tomato ALKBHs is shown. Included in the tree are all ALKBHs from *Arabidopsis* and two well-characterized ALKBHs, human ALKBH5 and mouse ALKBH5. The phylogenetic tree was produced using MEGA (version 5.2). Bootstrap values from 1000 replications for each branch are shown. Tomato proteins are indicated in red. Species names are abbreviated as follows: At, *Arabidopsis thaliana*; Sl, *Solanum lycopersicum*; Hs, *Homo sapiens*; Mm, *Mus musculus*. The accession numbers are indicated in parentheses. **b** Heat map analysis showing the gene expression of *SlALKBH1-8* in wild-type (WT) fruit at 39 DPA and 42 DPA and *Cnr* fruit at 42 DPA by RNA-seq. Data are presented as the mean of three independent biological replicates. Asterisks indicate significant differences (*P < 0.05, **P < 0.01, ***P < 0.001; Student’s *t* test). NS, no significance; DPA, days post-anthesis; FPKM, fragments per kilobase of exon per million mapped fragments. **c** Gene expression of *SlALKBH2* in WT fruit at different ripening stages and *Cnr* fruit at 42 DPA as determined by quantitative RT-PCR analysis. The *ACTIN* gene was used as an internal control. Error bars represent the standard deviation of three independent experiments. Asterisks indicate significant differences (*P < 0.05, **P < 0.01, ***P < 0.001; Student’s *t* test)

Based on the sequence of m^6^A RNA demethylase (ALKBHs) in animal and *Arabidopsis* [26, 29], we searched for the ALKBH candidates in tomato genome. A total of eight *ALKBH* genes were identified by screening the Sol Genomics Network (SGN) tomato database. They were named *SlALKBH1* to *SlALKBH8* according to their location on the chromosomes (Additional file 9: Table S18). All the tomato ALKBHs contain a highly conserved AlkB domain (Additional file 1: Figure S5) with Fe (II) binding sites and alpha-ketoglutaramate binding sites (Additional file 1: Figure S6). Phylogenetic analysis indicated that some tomato ALKBHs shared high similarity with each other (Fig. 6a; Additional file 10: Table S19), such as SlALKBH3 and SlALKBH4, suggesting gene duplications. Three tomato ALKBHs (SlALKBH2, 3, and 4) exhibit high similarity with *Arabidopsis* ALKBHs (Fig. 6a), which have been demonstrated to participate in plant development and defense response [29, 52]. Transcriptome analysis indicated that, among the eight tomato *ALKBH* genes, only *SlALKBH2* increased dramatically during fruit ripening but declined in the *Cnr* mutant (Fig. 6b), and this was confirmed by quantitative RT-PCR analysis (Fig. 6c). These data suggest that *SlALKBH2*, which was chosen for further analysis, might be regulated by DNA methylation and involved in fruit ripening. It is noteworthy that the expression of the potential m^6^A RNA methyltransferase genes (*MAT1-3*) is not altered substantially during fruit ripening or in the *Cnr* mutant (Additional file 1: Figure S7).

### DNA methylation regulates *SlALKBH2* transcript in tomato fruit

**Fig. 7:**
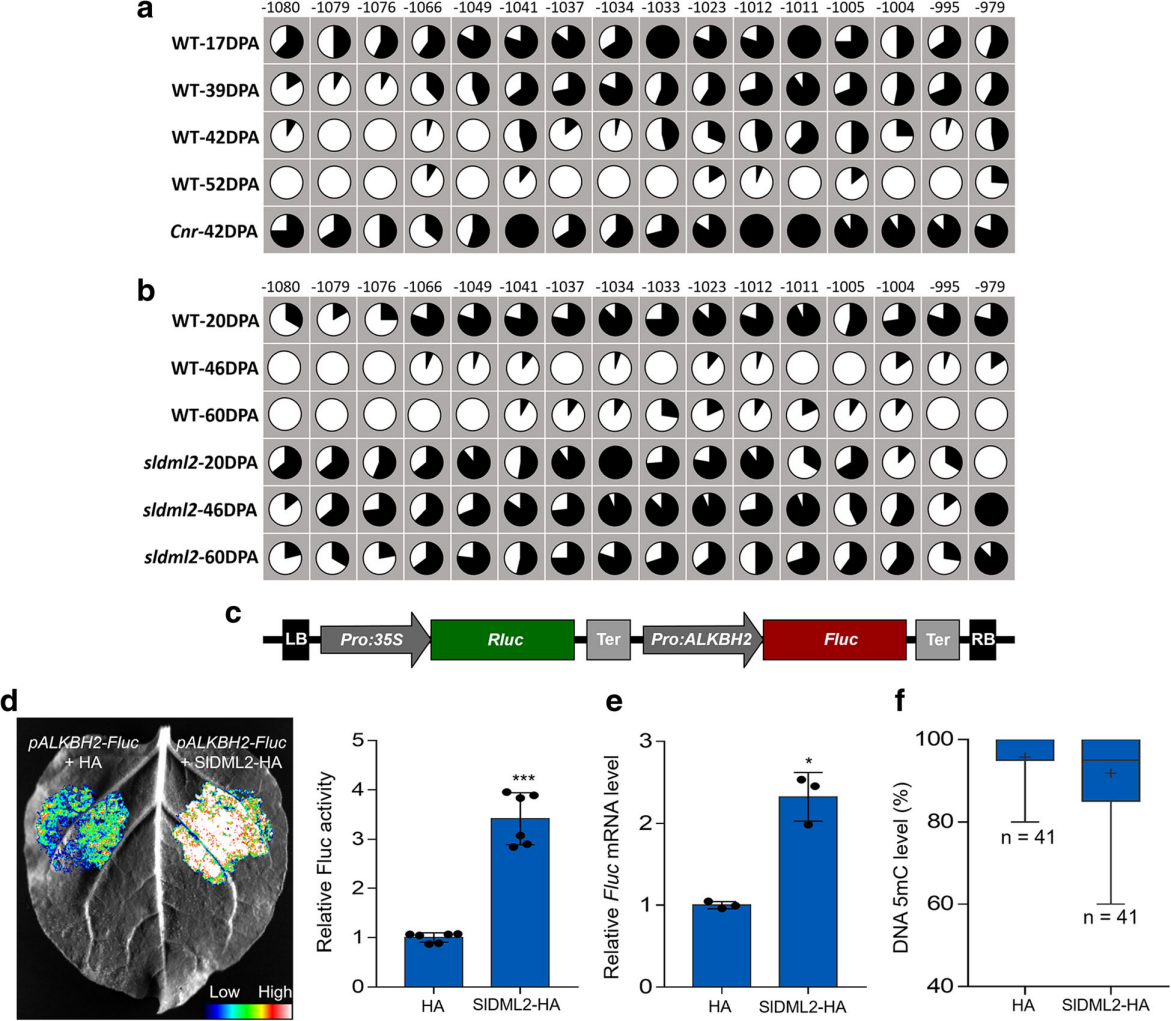
*SlALKBH2* is transcriptionally regulated by DNA methylation. **a** The 5mC levels in the differentially methylated region (DMR) of *SlALKBH2* promoter in wild-type (WT) and *Cnr* mutant fruit based on the Tomato Epigenome Database (http://ted.bti.cornell.edu/epigenome/). **b** The 5mC levels in the DMR of *SlALKBH2* promoter in WT and *sldml2* mutant fruit based on the DNA methylome database [38]. **a**, **b** The numbers indicate the cytosine positions relative to the start codon. Black represents the methylation frequency of cytosines at the indicated positions. DPA, days post-anthesis. **c** Schematic of the dual-luciferase system used for promoter activity assay. The *SlALKBH2* promoter was cloned into the dual-luciferase reporter vector to activate the expression of firefly luciferase (Fluc). The renilla luciferase (Rluc) driven by the CaMV 35S promoter served as an internal control. LB, left border; RB, right border; Ter, terminator. **d**–**f** Co-expression of SlDML2 (SlDML2-HA) with the dual-luciferase reporter vector in the *Nicotiana benthamiana* leaves increased the relative Fluc activity (**d**), facilitated the *Fluc* gene expression (**e**), and reduced the 5mC level in *SlALKBH2* promoter (**f**) compared with the empty plasmid control (HA). **d** The representative image from a total of six images (left panel). The Fluc activity was normalized against the Rluc activity, followed by normalization against the control (right panel). Data are presented as means ± standard deviation (*n* = 6). Asterisks indicate significant differences (***P < 0.0001; Student’s *t* test). **e** Gene expression was determined by quantitative RT-PCR analysis. Error bars represent the standard deviation of three independent experiments. Asterisks indicate significant differences (*P < 0.05; Student’s *t* test). **f** The box plot showing 5mC levels of all cytosines (*n* = 41) in the DMR analyzed by Sanger bisulfite sequencing. The plus sign represents the average level in each box

To determine whether *SlALKBH2* expression is regulated by DNA methylation, we examined the changes in DNA methylation patterns in *SlALKBH2* promoter during fruit ripening, as well as in the *Cnr* mutant, using the Tomato Epigenome Database (http://ted.bti.cornell.edu/epigenome/). A differentially methylated region (DMR) was found in the 5′ region of *SlALKBH2* at 979–1080 bp upstream of the start codon (Fig. 7a). This DMR becomes demethylated during ripening but remains hypermethylated in the fruit of *Cnr* mutant (Fig. 7a; Additional file 1: Figure S8). Interestingly, the hypermethylation of *SlALKBH2* promoter was also observed in the fruit of *sldml2* mutant (Fig. 7b), suggesting that *SlDML2* might participate in the regulation of *SlALKBH2* DNA demethylation.

To confirm that *SlALKBH2* transcription is regulated by DNA methylation, the promoter activity of *SlALKBH2* was assessed with a transient expression system in *Nicotiana benthamiana*. The *SlALKBH2* promoter was cloned into the dual-luciferase reporter plasmid (Fig. 7c), which contains a firefly luciferase (Fluc) reporter gene and a renilla luciferase (Rluc) reference gene. We found that, although weaker than the CaMV 35S promoter, the *SlALKBH2* promoter has the ability to activate Fluc expression (Additional file 1: Figure S9). The relative Fluc activity (Fig. 7d) and *Fluc* transcript level (Fig. 7e) were increased when *SlDML2* was co-expressed with the dual-luciferase reporter plasmid, concomitant with a decline in DNA methylation level of *SlALKBH2* promoter (Fig. 7f). Together, these data demonstrated that *SlALKBH2* transcription is regulated by DNA methylation and SlDML2 is involved in this process.

### SlALKBH2 is an active m^6^A RNA demethylase that locates in the endoplasmic reticulum

**Fig. 8:**
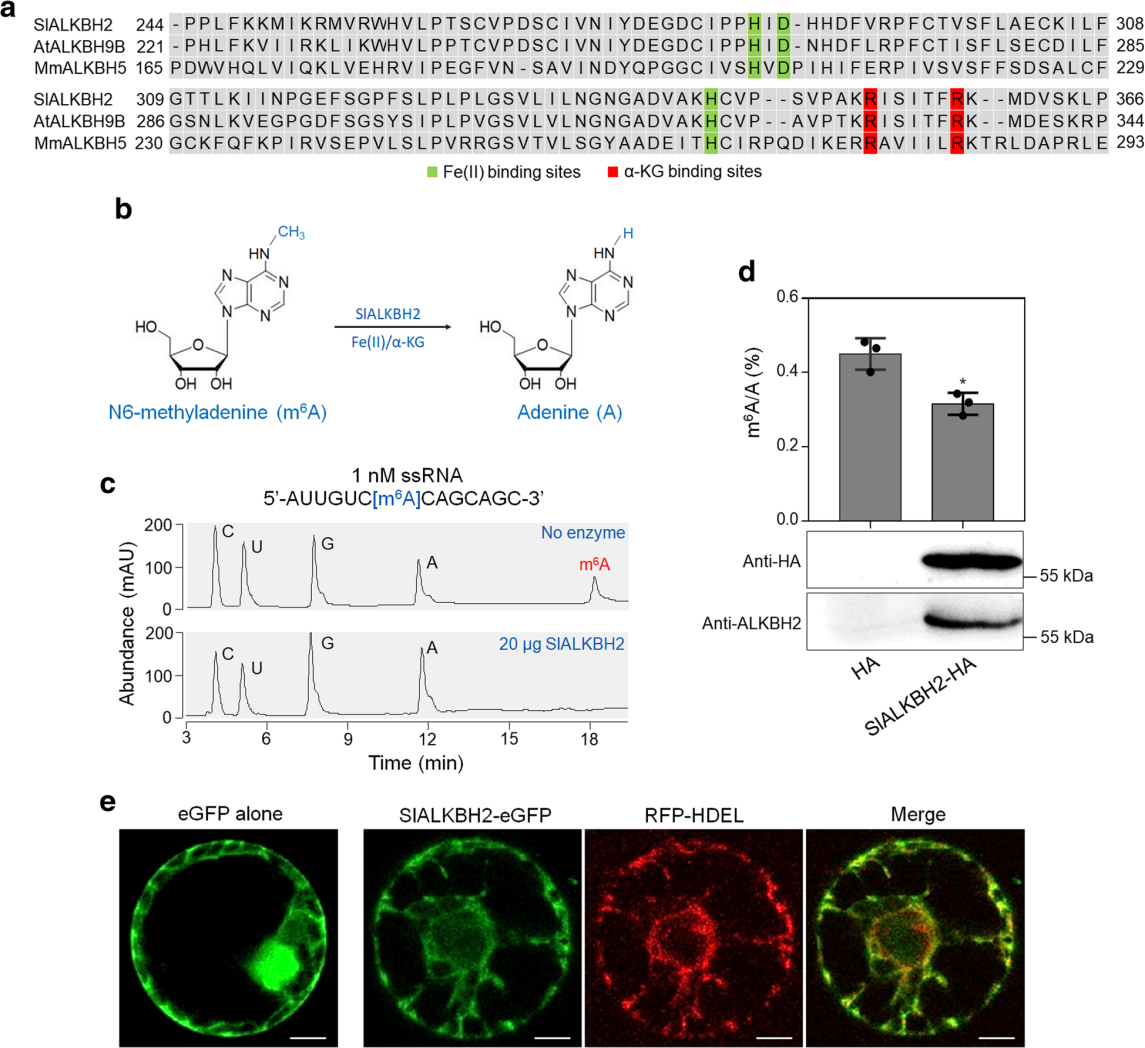
SlALKBH2 is a demethylase for mRNA m^6^A demethylation in tomato. **a** Sequence alignment of the highly conserved AlkB domain in SlALKBH2, *Arabidopsis* ALKBH9B (AtALKBH9B), and mouse ALKBH5 (MmALKBH5B). Fe (II) binding sites and alpha-ketoglutaramate (α-KG) binding sites are highlighted by green and red rectangles, respectively. **b** A proposed reaction mechanism of oxidative demethylation of N^6^-methyladenosine (m^6^A) to adenosine (A) by SlALKBH2. **c** Recombinant SlALKBH2 protein directly demethylates the m^6^A modification in m^6^A-containing ssRNA in vitro. The digested substrates cytidine (C), uridine (U), guanosine (G), A, and m^6^A were analyzed by HPLC. **d** SlALKBH2 demethylates m^6^A modification in mRNA in vivo. Endogenous mRNA was isolated from *Nicotiana benthamiana* leaves transiently expressing the SlALKBH2-HA fusion protein or the empty plasmid control (HA) and used for LC-MS/MS assay. Data are presented as mean ± standard deviation (*n* = 3). Asterisks indicate significant differences (*P < 0.05; Student’s *t* test). Immunoblot analysis was performed to detect SlALKBH2 expression using both anti-HA and anti-ALKBH2 antibodies. **e** Subcellular localization showing that SlALKBH2 locates in the endoplasmic reticulum (ER). His-Asp-Glu-Leu (HDEL) represents an ER retention signal peptide. Protoplasts of the *N. benthamiana* leaves transiently expressing eGFP alone or co-expressing ALKBH2-eGFP and HDEL-RFP were isolated and observed under a Leica confocal microscope (Leica, DMI600CS). Scale bar = 50 μm

Sequence alignment revealed that SlALKBH2 contains a highly conserved AlkB domain as that of *Arabidopsis* ALKBH9B (AtALKBH9B) and mouse ALKBH5 (MmALKBH5B) (Fig. 8a). To examine whether SlALKBH2 acts as an active m^6^A demethylase for oxidative demethylation of m^6^A to adenosine (A) (Fig. 8b), full-length SlALKBH2 was expressed in *Escherichia coli* as fusion proteins with a His-tag. The recombinant proteins were purified and used for demethylation assay using a synthetic 14 nucleotide-long m^6^A-modified ssRNA as a substrate (Fig. 8c). High-performance liquid chromatography (HPLC) analysis of the nucleosides digested from the substrate indicated that almost all of the methyls in m^6^A were effectively removed by recombinant SlALKBH2 in vitro (Fig. 8c), demonstrating that SlALKBH2 exhibited strong demethylation activity toward m^6^A in vitro.

To further verify the demethylation activity of SlALKBH2, the *SlALKBH2* CDS was fused with a HA-tag and transiently expressed in *N. benthamiana* leaves. Immunoblot analysis showed that *SlALKBH2* was successfully expressed (Fig. 8d). Detection of the overall mRNA m^6^A levels by LC-MS/MS indicated that the expression of *SlALKBH2* led to reduced m^6^A levels compared with the control (empty plasmid; Fig. 8d), indicating that SlALKBH2 possesses m^6^A demethylation activity.

To determine the intracellular localization of SlALKBH2, its CDS was introduced into a plasmid to generate a translational fusion with an enhanced green fluorescent protein (eGFP) at the C-terminus. The construct was agroinfiltrated into the *N. benthamiana* leaves, and then the mesophyll protoplasts were isolated and used for fluorescence microscopy. Confocal laser scanning microscopy showed that eGFP-tagged SlALKBH2 (SlALKBH2-eGFP) displayed a strong signal in the endoplasmic reticulum (ER), while the eGFP-only control produced a fluorescent signal throughout the cell, except the vacuolar lumen (Fig. 8e). The fluorescent signals of SlALKBH2-eGFP co-localized with those of His-Asp-Glu-Leu (HDEL)-tagged red fluorescent protein (RFP-HEDL), which was used as a marker for ER location [53], confirming the intracellular localization of SlALKBH2 in ER (Fig. 8e).

### SlALKBH2-mediated m^6^A demethylation stabilizes *SlDML2* mRNA

**Fig. 9:**
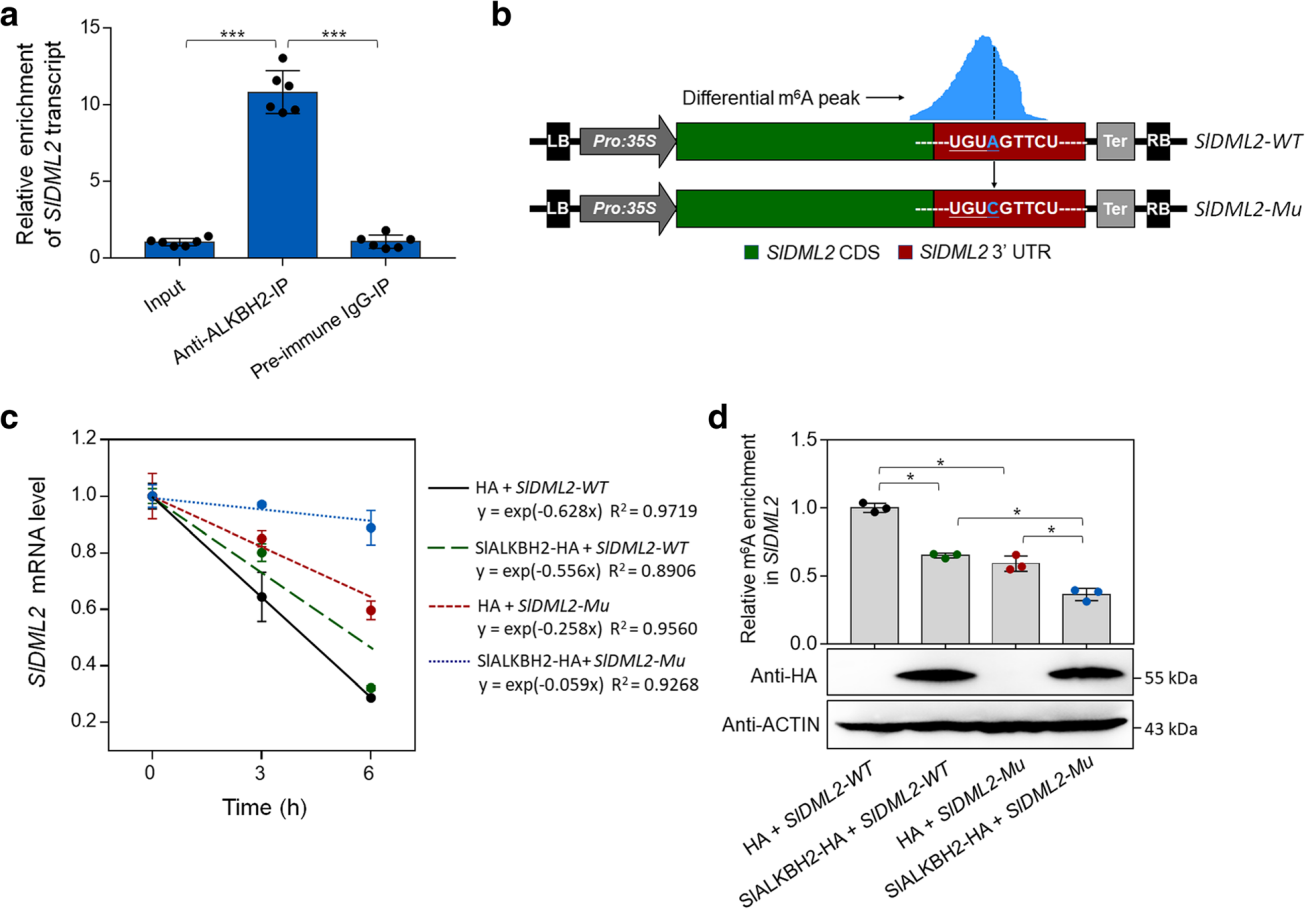
SlALKBH2 protein binds *SlDML2* transcript and promotes its stability by m^6^A demethylation. **a** RNA immunoprecipitation (RIP) assay showing that SlALKBH2 protein binds *SlDML2* transcript. For the RIP assay, the protein-RNA complexes were extracted from wild-type tomato fruit at 42 days post-anthesis and subjected to immunoprecipitation with anti-ALKBH2 polyclonal antibody or rabbit IgG (negative control). Data are presented as mean ± standard deviation (*n* = 6). Asterisks indicate significant differences (***P < 0.001; Student’s *t* test). **b** Schematic of the transient expression system used for *SlDML2* mRNA stability assay. The intact or mutated *SlDML2* cDNA fragment, which is composed of coding sequence (CDS) and 3′ untranslated region (UTR), was cloned into pCambia2300 vector driven by the CaMV 35S promoter. The potential m^6^A modification site identified in m^6^A-seq was mutated from adenosine (A) to cytidine (C) using site-directed mutagenesis kit and highlighted in blue. **c** Determination of the *SlDML2* mRNA stability. The intact (*SlDML2-WT*) or mutated *SlDML2* (*SlDML2-Mu*) cDNA fragment was co-expressed with SlALKBH2-HA or empty vector control (HA) in *Nicotiana benthamiana* leaves. After actinomycin D treatment for 3 or 6 h, the total RNAs were isolated from the *N. benthamiana* leaves and submitted to quantitative RT-PCR assay. The *N. benthamiana ACTIN* gene was used as an internal control. Error bars represent the standard deviation of three independent experiments**. d** m^6^A-IP-qPCR assay showing the m^6^A enrichment in *SlDML2* transcript. Data are presented as mean ± standard deviation (*n* = 3). Immunoblot analysis with anti-HA and anti-ALKBH2 antibodies shows the SlALKBH2 protein expression. Asterisks indicate significant differences (*P < 0.05; Student’s *t* test)

We next sought to explore whether SlALKBH2 could directly bind to mRNAs of *SlDML2*, *NR*, and *FUL2*, which show differential m^6^A methylation in our m^6^A-seq analyses (Fig. 5a), using RNA immunoprecipitation (RIP). A polyclonal antibody that specifically recognized SlALKBH2 (Additional file 1: Figure S10) was used to immunoprecipitate SlALKBH2-bound mRNAs, and the result revealed a direct interaction between SlALKBH2 and *SlDML2* transcript (Fig. 9a). No interaction between SlALKBH2 and *NR* or *FUL2* transcript was observed (Additional file 1: Figure S11), indicating that the m^6^A mRNA demethylation of these two genes was mediated by other components of the m^6^A pathway instead of SlALKBH2.

m^6^A methylation has been demonstrated to decrease mRNA stability, especially when m^6^A is located at the stop codon or 3′ UTR [6, 10, 29, 49]. As *SlDML2* exhibits m^6^A modification within the 3′ UTR, we set out to determine if the m^6^A methylation affects *SlDML2* mRNA stability. The cDNA fragment of *SlDML2* composed of CDS and 3′ UTR was introduced into pCambia2300 vector (Fig. 9b), which was subsequently agroinfiltrated into the *N. benthamiana* leaves for transient expression. The *SlDML2* mRNA stability was measured by monitoring the degradation rate of mRNA after treatment with transcription inhibitor actinomycin D. As shown in Fig. 9c, *SlDML2* mRNA degraded quickly after actinomycin D treatment. When *SlALKBH2* was co-expressed with *SlDML2* in *N. benthamiana*, the degradation rate of *SlDML2* mRNA decreased, concomitant with a significant decrease in m^6^A abundance of *SlDML2* (Fig. 9d). Importantly, a mutated form of *SlDML2* in which the potential m^6^A modification site identified in m^6^A-seq was mutated from A to C (Fig. 9b) degraded slower than the intact *SlDML2* (Fig. 9c). Co-expression of the mutated form of *SlDML2* with *SlALKBH2* further decreased the degradation rate of *SlDML2* mRNA (Fig. 9c). Together, these data suggest that m^6^A modification promotes mRNA degradation of *SlDML2*, and SlALKBH2-mediated m^6^A demethylation stabilizes *SlDML2* mRNA.

### SlALKBH2 is required for normal tomato fruit ripening

**Fig. 10:**
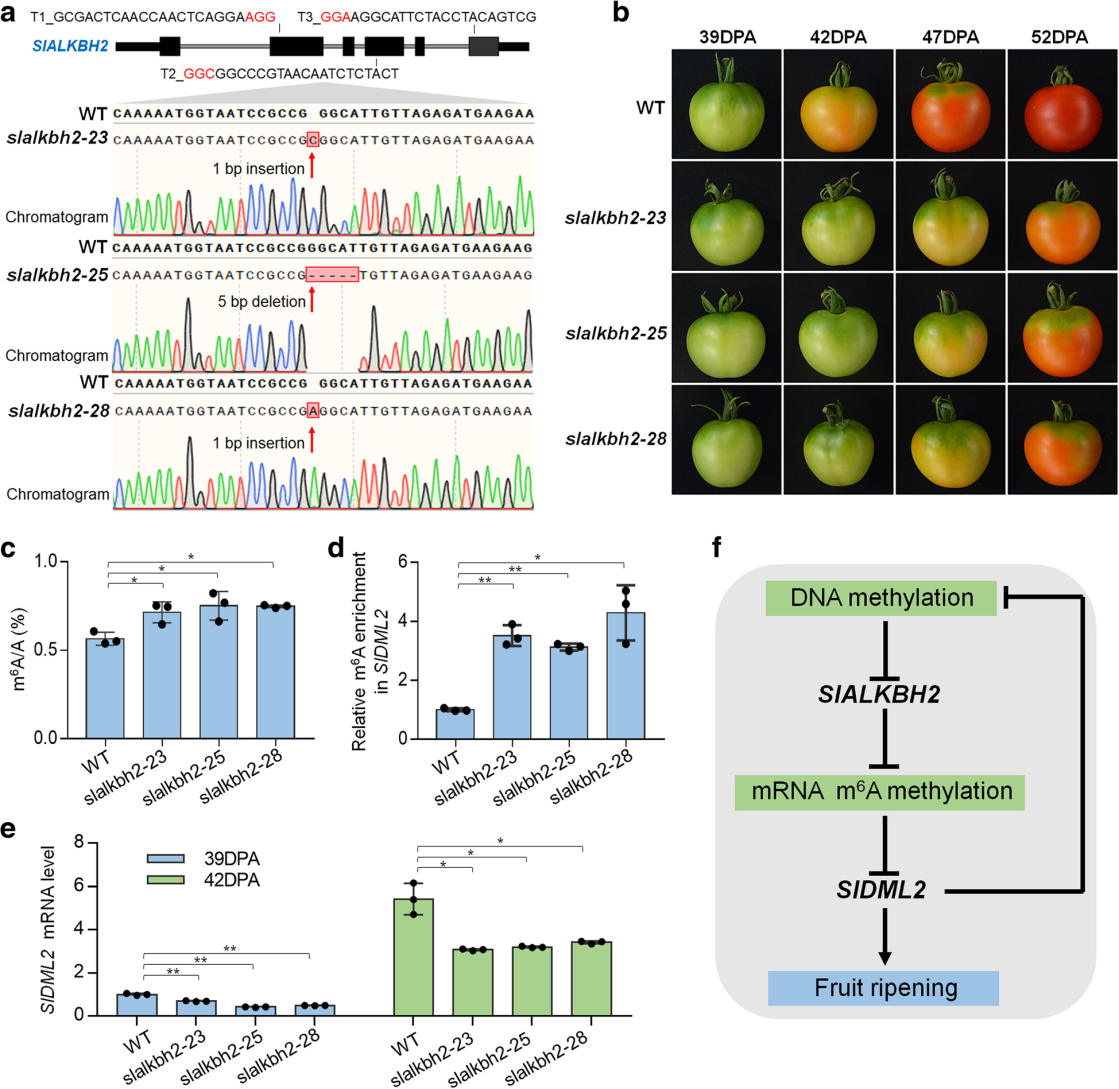
SlALKBH2 is necessary for normal tomato fruit ripening. **a** Genotyping of mutations mediated by CRISPR/Cas9 gene-editing system in *slalkbh2-23*, *slalkbh2-25*, and *slalkbh2-28* mutants. Diagram showing the single guide RNAs (sgRNAs) containing different target sequences (T1, T2, and T3), which were designed to specifically target the exons of *SlALKBH2*. The red letters indicate the protospacer adjacent motif (PAM). The transgenic plants in the second generation were genotyped by sequencing genomic regions flanking the target sites. Red arrows indicate the editing sites. Two mutants (*slalkbh2-23* and *slalkbh2-28*) have a homozygous 1-bp insertion, and one (*slalkbh2-25*) has a homozygous 5-bp deletion caused by target T2 in the fourth exon of *SlALKBH2*. **b** Ripening phenotype of *slalkbh2* mutants. Fruit from wild-type (WT) and *slalkbh2* mutants (*slalkbh2-23*, *slalkbh2-25*, and *slalkbh2-28*) at 39, 42, 47, and 52 days post-anthesis (DPA) are shown. **c** LC-MS/MS assay showing the amount of mRNA m^6^A in WT and *slalkbh2* mutant fruit at 39 DPA. Data are presented as mean ± standard deviation (*n* = 3). **d** m^6^A-IP-PCR assay showing the relative m^6^A enrichment in *SlDML2* mRNA in WT and *slalkbh2* mutant fruit at 39 DPA. **e** *SlDML2* gene expression in WT and *slalkbh2* mutant fruit at 39 and 42 DPA. The *ACTIN* gene was used as an internal control. **d**, **e** Error bars represent the standard deviation of three independent experiments. Asterisks indicate significant differences (*P < 0.05, **P < 0.01; Student’s *t* test). **f** Model for the relationship between DNA methylation and m^6^A mRNA methylation in fruit ripening. DNA methylation negatively regulates *SlALKBH2* to mediate overall m^6^A mRNA methylation. The m^6^A modification promotes *SlDML2* mRNA decay, thereby affecting DNA methylation and fruit ripening

We subsequently examine if SlALKBH2 influences tomato fruit ripening using a CRISPR/Cas9 gene-editing system. Three single guide RNAs (sgRNAs) containing different target sequences (T1, T2, and T3) were designed to specifically target the exons of *SlALKBH2* (Fig. 10a). These sgRNA sequences were cloned into a binary vector that harbors Cas9 expression cassettes [54], and the resulting construct was transformed into wild-type tomato in the cv. Ailsa Craig background using *Agrobacterium* infection of leaf explants [55, 56]. Among transgenic plants in the second generation, we isolated three distinct homozygous mutant lines (*slalkbh2-23*, *slalkbh2-25*, and *slalkbh2-28*) through direct sequencing of PCR products from genomic DNA flanking the target sites. These homozygous mutants carry 1-bp insertion (*slalkbh2-23* and *slalkbh2-28*) or 5-bp deletion (*slalkbh2-25*) caused by target T2 in the fourth exon of *SlALKBH2* (Fig. 10a), and no editing events were found around the sequence of target T1/3. All mutants were predicted to cause premature stop codon within the following 10-bp sequence of editing sites. We did not find any off-target editing events in the seven potential off-target genes that were predicted by CRISPR-P (version 2.0, http://crispr.hzau.edu.cn/CRISPR2/) (Additional file 1: Figure S12).

By comparing the fruit of the wild-type and *slalkbh2* mutants at 39, 42, 47, and 52 DPA, we found that *slalkbh2-23*, *slalkbh2-25*, and *slalkbh2-28* mutant lines showed similar and obvious ripening-delayed phenotypes (Fig. 10b). A visible color change was observed at 42 DPA in the wild-type fruit, whereas the *slalkbh2* mutant tomatoes remained green at this stage (Fig. 10b). At 47 DPA, the wild-type fruit had a homogenous orange color, while the fruit from the *slalkbh2* mutants was only just starting to change color. This indicates that *SlALKBH2* is indispensable for normal tomato fruit ripening. LC-MS/MS was subsequently performed to assay the total mRNA m^6^A levels in the wild type and *slalkbh2* mutants at 39 DPA, and the result indicated that mutation of *SlALKBH2* led to a significantly higher mRNA m^6^A levels (Fig. 10c). Meanwhile, the m^6^A-IP-qPCR assay showed that *slalkbh2* mutants exhibited higher m^6^A abundance in the transcript of *SlDML2* compared to the wild type at this stage (Fig. 10d). By contrast, the mRNA level of *SlDML2* declined in the *slalkbh2* mutants (Fig. 10e). These data reveal that SlALKBH2 is necessary for m^6^A regulation during tomato fruit ripening. SlALKBH2 might participate in the regulation of fruit ripening by modulating *SlDML2* mRNA stability through m^6^A demethylation. Notably, the regulation of m^6^A is complicated, and other factors in addition to *SlALKBH2* might play roles in this process.

## Discussion

DNA methylation has been elucidated to play an essential role in the regulation of fruit ripening [35–39]. It is unclear whether mRNA m^6^A modification, which is considered as an mRNA “epitranscriptome” [3, 57], participates in this process. In the present study, we show that m^6^A methylation represents a widespread mRNA modification in tomato and correlates with fruit ripening. The m^6^A modification is primarily located around the stop codon and within the 3′ UTR of coding genes, and the sequence motif was conserved with that in *Arabidopsis*. The mRNA m^6^A methylation in tomato fruit is mediated by endoplasmic reticulum-located m^6^A RNA demethylase SlALKBH2 during ripening. We demonstrate that DNA methylation regulates the transcription of *SlALKBH2*, which in turn functions on m^6^A demethylation of *SlDML2* mRNA and modulates its stability. Our findings uncover the interplay between DNA and RNA methylation and reveal a novel layer of gene regulation in fruit ripening.

### m^6^A RNA demethylase gene *SlALKBH2* is regulated by DNA methylation and required for normal fruit ripening

m^6^A modification could be dynamically regulated by both RNA methyltransferases and demethylases, which catalyze the m^6^A formation and removal, respectively [3, 9, 15]. Substantial insights have been made into the physiological functions of RNA methyltransferases in mammals and plants [6, 18, 29, 30, 58]. Furthermore, a recent study unveiled that the activity of RNA methyltransferase METTL3 in mammals was regulated by SUMOylation [59]. By contrast, the biological importance and the regulatory mechanisms underlying RNA demethylation remain largely unknown. In the model plant *Arabidopsis*, there are five potential RNA demethylases, among which ALKBH10B functions in floral transition [29], while ALKBH9B modulates infection of alfalfa mosaic virus [52]. We carried out an extensive search of the tomato genome and identified eight putative RNA demethylases (SlALKBH1 to 8) that contain AlkB domain. Gene expression analysis indicated that *SlALKBH2* increased dramatically in ripening fruit that undergoes genome-wide loss of DNA methylation but declined in the fruit of ripening-deficient mutant *Cnr* that displays DNA hypermethylation (Fig. 6). This negative correlation between *SlALKBH2* transcription and DNA methylation status led us to speculate that *SlALKBH2* is regulated by DNA methylation. As expected, we found that the promoter region of *SlALKBH2* contains an obvious differentially methylated region (DMR) and demethylation of *SlALKBH2* increased its transcript level (Fig. 7). Further analysis indicated that SlALKBH2 possesses RNA demethylation activity (Fig. 8) and mutation of *SlALKBH2* delays fruit ripening (Fig. 10). Notably, DMRs were also found in the promoters of other putative RNA demethylase genes (Additional file 1: Figure S8), but the transcription of these genes changes slightly during fruit ripening (Fig. 6), suggesting that they might be dispensable for tomato ripening.

The primary function of DNA methylation was thought to regulate gene expression at transcriptional level [40–42]. However, recent researches revealed that DNA methylation could also impact gene expression at post-transcriptional level via regulation of mRNA alternative splicing [43, 44]. Moreover, it was demonstrated that some long non-coding RNA (lncRNA) promoters were targeted by DNA methylation [60], indicating that DNA methylation could regulate gene expression at multiple levels directly or indirectly. Our observation that *SlALKBH2* was regulated by DNA methylation revealed that DNA methylation could impact gene expression through regulation of mRNA m^6^A modification.

### Modulation of DNA demethylase gene *SlDML2* by SlALKBH2-mediated m^6^A demethylation

m^6^A methylation affects gene expression by modulation of RNA metabolism [8–15]. It was reported that m^6^A methylation negatively affects the stability of target mRNAs and subsequent protein synthesis, thus acting as a negative regulator of gene expression [6, 10, 29, 49]. In the m^6^A RNA methylome analyses, we found that hundreds of ripening-induced and ripening-repressed genes showed differential m^6^A levels between the samples (39 DPA wild type vs. 42 DPA wild type or 42 DPA wild type vs. 42 DPA *Cnr* mutant) (Additional file 8: Table S16-S17), and m^6^A deposition usually correlated with the decrease in gene expression (Fig. 4). Interestingly, the transcript of *SlDML2*, a DNA demethylase gene [37], exhibited higher m^6^A level in the fruit of *Cnr* mutant, concomitant with a decline in *SlDML2* mRNA level, compared with the wild type (Fig. 5). This suggests that m^6^A methylation might participate in the regulation of *SlDML2* mRNA abundance. To verify this speculation, we firstly assessed whether m^6^A modification promotes *SlDML2* mRNA degradation. We found that the degradation rate of *SlDML2* mRNA was decreased when the m^6^A sites were mutated (Fig. 9)*.* Furthermore, the RNA demethylase SlALKBH2, which binds *SlDML2* mRNA and mediates its m^6^A demethylation, could stabilize *SlDML2* mRNA (Fig. 9). We then mutated *SlALKBH2* and observed that mutation of *SlALKBH2* led to the increase in m^6^A level of *SlDML2* transcript, accompanied by the decline in *SlDML2* mRNA level (Fig. 10). Together, these findings indicated that the mRNA abundance of *SlDML2* was regulated by SlALKBH2-mediated m^6^A demethylation.

As a dynamic modification, the status of DNA methylation is enzymatically controlled by the combined actions of methylation and demethylation reactions that introduce and remove this mark, respectively [40]. In plants, active DNA demethylation is initiated by a subfamily of bifunctional 5-methylcytosine DNA glycosylases/lyases that include *Arabidopsis* proteins repressor of silencing 1 (ROS1) [61], Demeter (DME), and Demeter-like proteins 2 and 3 (DML2 and DML3) [62–64]. Recently, active DNA demethylation was revealed to be regulated by an RNA-binding protein ROS3 and a histone acetyltransferase IDM1 that are required for the recruitment of ROS1 to the chromatin [65, 66]. However, it remains uncertain whether DNA demethylation is regulated at post-transcriptional level. Data from this study provide evidence that *SlDML2*, the close homolog of the *Arabidopsis* DNA demethylase gene *ROS1* [37, 38], is regulated by SlALKBH2-mediated m^6^A modification. SlDML2 was reported to be responsible for ripening-induced DNA demethylation in tomato [38]. Hundreds of ripening-related genes could be activated by SlDML2, and loss-of-function *sldml2* mutant exhibits dramatic inhibition of fruit ripening [38]. Considering the importance of SlDML2 in fruit ripening, we suggest that SlALKBH2 regulates ripening, at least partially, by targeting *SlDML2* and mediating its mRNA stability. It should be noted that SlALKBH2 might influence fruit ripening by concurrently targeting transcripts of other ripening-related genes. The SlALKBH2-mediated m^6^A modification of these transcripts and their molecular link to fruit ripening deserve further research. Based on our results and previous studies, we propose a model for the correlation between DNA methylation and m^6^A mRNA methylation in fruit ripening (Fig. 10f).

In conclusion, our findings reveal that DNA methylation regulates m^6^A methylation by targeting RNA demethylase gene *SlALKBH2*, which in turn influences DNA methylation via DNA demethylase gene *SlDML2* by a feedback loop to affect fruit ripening. Considering the multiple roles of DNA methylation and m^6^A methylation, the regulation we describe here may have an essential function in many cellular contexts.

## Methods

### Plant materials

Seeds of tomato (*Solanum lycopersicum* cv. Ailsa Craig), including wild type and the ripening-deficient mutant *Colorless non-ripening* (*Cnr*) in the cv. Ailsa Craig background, were obtained from the Tomato Genetics Resource Center (TGRC, https://tgrc.ucdavis.edu/policy.aspx). The plants were grown under standard culture conditions in a greenhouse, which was supplied with regular fertilizer and supplementary lighting when required. Flowers were tagged at the anthesis to accurately determine the age of fruit through development and ripening. Wild-type fruit were harvested at immature green (IM), mature green (MG), breaker (Br), orange ripe (OR), and red ripe (RR), which were on average 17, 39, 42, 47, and 52 days post-anthesis (DPA), respectively, based on the size, shape, color, and the development of seed and locular jelly in the fruit [67]. The fruit of *Cnr* and *slalkbh2* mutants were harvested at the equivalent ripening stages, as determined by the DPA. The pericarp tissues were collected immediately after harvesting, frozen in liquid nitrogen, and then stored at − 80 °C until use.

### Global DNA methylation assay

Global 5mC levels in tomato genomic DNA was determined as previously described with minor modifications [68]. In brief, DNA was extracted from the pericarp tissues using Sureplant DNA kit (Cwbiotech, CW2298), with the disruption of total RNA according to the manufacturer’s protocols. The extracted DNA was detected in 1% agarose gel and quantified by a SimpliNano spectrophotometer (GE Healthcare, 29-0617-11). Then, 100 ng of purified and integrated DNA for each measurement was used to perform 5mC assay by MethylFlash™ methylated DNA quantification kit (Epigentek, P-1034). 5mC levels in different DNA samples were relatively quantified using both the negative control and positive control, which contain 0% 5mC and 50% 5mC, respectively, following the manufacturer’s instructions.

### Quantitative analysis of mRNA m^6^A by LC-MS/MS

Total RNAs were extracted from tomato pericarps or *N. benthamiana* leaves following the method of Moore et al. [69]. mRNAs were isolated from total RNAs by using Dynabeads mRNA purification kit (Life Technologies, 61006). Two hundred nanograms of mRNAs was digested with 1 unit of Nuclease P1 (Wako, 145-08221) in 50 μL reaction buffer (10 mM ammonium acetate, pH 5.3, 25 mM NaCl, 2.5 mM ZnCl_2_) at 37 °C for 6 h. Then, 5.5 μL 1 M fresh NH_4_HCO_3_ and 1 unit of alkaline phosphatase (Sigma-Aldrich, P6774) were added and incubated at 37 °C for another 6 h. The digested samples were centrifuged at 15,000*g* for 5 min, and the supernatants were used to LC-MS/MS analysis. The nucleosides were separated by UPLC (Waters, ACQUITY) equipped with a ACQUITY UPLC HSS T3 column (Waters) and detected by MS/MS using a Triple Quad Xevo TQ-S (Waters) mass spectrometer in positive ion mode by multiple reaction monitoring. The mobile phase consists of buffer A (5 mM ammonium acetate) and buffer B (100% acetonitrile). Nucleosides were quantified using the nucleoside-to-base ion mass transitions of m/z 268.0 to 136.0 (A) and m/z 282.0 to 150.1 (m^6^A). Standard curves were generated by running a concentration series of pure commercial A (TargetMol, T0853) and m^6^A (TargetMol, T6599). Contents of nucleosides in samples were calculated by fitting the peak areas to the standard curves. The m^6^A/A ratio was calculated accordingly. The experiment was performed with three independent biological replicates.

### m^6^A-seq

The m^6^A-seq was performed as previously described [45]. Briefly, total RNAs were extracted from the pericarp tissues of wild-type fruit at 39 DPA and 42 DPA and *Cnr* fruit at 42 DPA. The integrity and concentration of extracted RNAs were detected by using an Agilent 2100 bioanalyzer (Agilent, G2939A) and a SimpliNano spectrophotometer (GE Healthcare, 29-0617-11), respectively. Then, mRNAs were isolated from intact total RNAs using Dynabeads mRNA purification kit (Life Technologies, 61006) and fragmented into ~ 100 nucleotide-long fragments by incubation for 5 min at 94 °C in the RNA fragmentation buffer (10 mM Tris-HCl, pH 7.0, 10 mM ZnCl_2_). The fragmentation reaction was stopped by the addition of 50 mM EDTA, and then the fragmented mRNAs were purified by phenol-chloroform extraction and ethanol precipitation.

For the m^6^A-seq, 5 μg of fragmented mRNAs was incubated with 10 μg of anti-m^6^A polyclonal antibody (Synaptic Systems, 202003) at 4 °C for 2 h in 450 μL of immunoprecipitation (IP) buffer containing 10 mM Tris-HCl, pH 7.4, 150 mM NaCl, 0.1% NP-40 (*v*/*v*), and 300 U mL^−1^ RNase inhibitor (Promega, N2112S). The mixture was then immunoprecipitated by incubation with 50 μL of Dynabeads Protein-A (Life Technologies, 10002A) at 4 °C for another 2 h. After washing twice with high-salt buffer consisting of 50 mM Tris-HCl, pH 7.4, 1 M NaCl, 1 mM EDTA, 1% NP-40 (*v*/*v*), and 0.1% SDS (*w*/*v*) and twice with IP buffer, the bound mRNAs were eluted from the beads by incubation with 6.7 mM N^6^-methyladenosine (Sigma, M2780) in IP buffer and recovered with phenol-chloroform extraction and ethanol precipitation. Then, 50 ng of immunoprecipitated mRNAs or pre-immunoprecipitated mRNAs (input control) was used for library construction with NEBNext ultra RNA library prepare kit for Illumina (NEB, E7530). High-throughput sequencing was performed on the Illumina HiSeq X sequencer with a paired-end read length of 150 bp according to the standard protocols. The sequencing was carried out with three independent biological replicates, and each RNA sample was prepared from the mix of at least 30 tomato fruits to avoid individual difference among fruits.

### m^6^A-seq data analysis

The quality of raw sequencing reads in m^6^A-seq was assessed using FastQC tool (version 0.11.7) [70]. Adaptors and low-quality bases with a score < 20 located in the 3′-end were trimmed from all raw reads by Cutadapt software (version 1.16) [71]. After trimming, reads containing ambiguous nucleotides or with a length < 18 nucleotides were filtered out by Trimmomatic (version 0.30) [72]. The remaining reads were analyzed by using FastQC tool once again to ensure sufficient quality assessment. Then, read alignment was performed with Burrows Wheeler Aligner (BWA; version 0.30) [73] by using the tomato build_SL3.0 as a reference genome, and the ITAG3.2_release as a reference annotation (ftp://ftp.solgenomics.net/tomato_genome/). Mapping quality (MAPQ) of all aligned reads was concurrently calculated, and only uniquely mapped reads with a MAPQ ≥ 13 were remained for the subsequent analysis for each sample [45].

MACS software (version 2.0.10) [74] was used for the m^6^A peak identification in each anti-m^6^A immunoprecipitation sample with the corresponding input sample serving as a control. A stringent cutoff threshold for MACS-assigned false discovery rate (FDR) < 0.05 was used to obtain high-confidence peaks. Only the peaks consistently called in all three independent biological samples were considered as confident peaks and used for subsequent analysis. PeakAnnotator (version 2.0) [75] was applied to annotate confident peaks to the tomato ITAG3.2_release annotation file. Differentially methylated peaks between the samples were determined using the m^6^A site differential algorithm [76] with a criterion of *P* value < 0.05 and enrichment fold change ≥ 1.5. The m^6^A-enriched motifs were identified by HOMER (version 4.7; http://homer.ucsd.edu/homer/) [47]. All peaks mapped to mRNAs were used as the target sequences, and the exon sequences except for the peak-containing sequences were used as the background sequences. The motif length was restricted to six nucleotides. Visualization analysis of m^6^A peaks was carried out using Integrated Genome Browser (IGB, version 9.0.2) [77]. Gene Ontology (GO) analysis of m^6^A-modified genes was performed on Gene Ontology Consortium (http://www.geneontology.org/). GO term with a Bonferroni-corrected *P* value < 0.05 in individual genes was considered to be statistically significant.

### RNA-seq

The input sequencing reads in the m^6^A-seq were used for RNA-seq analysis as previously described [29]. Briefly, the uniquely mapped reads of each sample were assembled by Cufflinks [78]. Gene expression was calculated as fragments per kilobase of exon per million mapped fragments (FPKM) by using Cuffdiff, which concurrently provides statistical routines for determining differential gene expression [78]. The resulting *P* values were adjusted using the Benjamini and Hochberg’s approach [79] for controlling the false discovery rate (FDR). Differentially expressed genes were defined based on a cutoff criterion of FPKM fold change ≥ 1.5 and *P* value < 0.05.

### m^6^A-IP-qPCR

m^6^A-IP-qPCR was performed as previously described with some modifications [80]. Briefly, 5 μg of purified mRNAs were fragmented into ~ 300 nucleotide-long fragments by 30 s incubation at 94 °C in the RNA fragmentation buffer (10 mM Tris-HCl, pH 7.0, 10 mM ZnCl_2_). The fragmentation reaction was stopped by the addition of 50 mM EDTA, followed by phenol-chloroform extraction and ethanol precipitation to purify the fragmented mRNAs. The fragmented mRNAs were resuspended in 250 μL DEPC-treated water; 5 μL was used as the input sample. Then, 100 μL of fragmented mRNAs were incubated with 5 μg of anti-m^6^A polyclonal antibody at 4 °C for 2 h in 450 μL of IP buffer containing 10 mM Tris-HCl, pH 7.4, 150 mM NaCl, 0.1% NP-40 (*v*/*v*), and 300 U mL^−1^ RNase inhibitor (Promega, N2112S). The mixture was then immunoprecipitated by incubation with 20 μL of Dynabeads Protein-A (Life Technologies, 10002A) at 4 °C for another 2 h. After washing twice with high-salt buffer containing 50 mM Tris-HCl, pH 7.4, 1 M NaCl, 1 mM EDTA, 1% NP-40 (*v*/*v*), and 0.1% SDS (*w*/*v*) and twice with IP buffer, the bound mRNAs were eluted from the beads by incubation with 6.7 mM N^6^-methyladenosine (Sigma, M2780) in IP buffer at 4 °C for 1 h and then recovered with phenol-chloroform extraction and ethanol precipitation. The immunoprecipitated mRNA fragments were resuspended in 5 μL DEPC-treated water. Then, the immunoprecipitated mRNA and pre-immunoprecipitated mRNA (input mRNA) were reverse transcribed with random hexamers using M-MLV reverse transcriptase (Takara, 2640A) and submitted to PCR amplification as quantitative RT-PCR below. m^6^A enrichment in specific gene regions was determined by using the cycle threshold (C_T_) 2^(−ΔCT)^ method [81]. The value for the immunoprecipitated sample was normalized against that for *ACTIN* (Solyc03g078400), which did not show any obvious mRNA m^6^A peak from m^6^A-seq data, as an internal control, and then normalized against that for the input. All primers used for m^6^A-IP-qPCR are listed in Additional file 11: Table S20. Each experiment has three biological replicates and each with three technical repeats.

### Quantitative RT-PCR analysis

Total RNAs were extracted from tomato pericarps or *N. benthamiana* leaves as described above. Extracted RNAs were treated with DNase I (Takara, D2215) and then used to synthesize cDNA by reverse transcription with an oligo (dT)_18_ primer using the Moloney murine leukemia virus (M-MLV) reverse transcriptase (Takara, 2640A). Quantitative RT-PCR was conducted on the StepOnePlus Real-Time PCR System (Applied Biosystems) using the SYBR green PCR master mix (Applied Biosystems, 4367659). PCR amplification with the gene-specific primers listed in Additional file 11: Table S21 was performed with the following program in a volume of 20 μL: 95 °C for 10 min, followed by 40 cycles of 95 °C for 15 s and 60 °C for 30 s. Relative mRNA levels were quantified by using the cycle threshold (C_T_) 2^(−ΔCT)^ method [81]. Tomato *ACTIN* (Solyc03g078400) or *N. benthamiana ACTIN* (Niben101Scf03410g03002) was used to normalize the expression values. Each experiment contained three biological replicates and each with three technical repeats.

### Tomato ALKBHs identification and phylogenetic analysis

For tomato AlkB homolog (ALKBHs) identification, the protein sequences of known *Arabidopsis* ALKBHs [29], human ALKBH5 [82], and mouse ALKBH5 [26] were used in BLAST searches against the Sol Genomics Network (SGN) tomato database (https://www.sgn.cornell.edu/) with default parameters. Obtained protein sequences were used for further searching under the same conditions to avoid omissions. The conserved domain of all identified sequences was analyzed on pfam (http://pfam.xfam.org/), and only the protein containing an AlkB domain (PF13532) was remained and considered as a tomato ALKBH. For phylogenetic analysis, the sequences of tomato ALKBHs (Additional file 1: Supplementary text) were aligned with the sequences of *Arabidopsis* ALKBHs, human ALKBH5, and mouse ALKBH5 using Clustal X software (version 2.1) with standard parameters. The alignment result was manually edited by the Genedoc program and then imported into MEGA software (version 5.2) to construct a phylogenetic tree using the neighbor-joining statistical method with 1000 bootstrap replicates.

### *SlALKBH2* promoter activity assay

For promoter activity assay, the *SlALKBH2* promoter fragment (~ 2000 bp upstream of the start codon) was cloned from tomato genomic DNA using the primers F (5′-GTTAACACATAAATGGTAGCTATTCAC-3′) and R (5′-CCTGATTTTAATTTCTCCGATCAAC-3′). The amplified fragment was inserted into the dual-luciferase reporter vector pGreenII-0800-LUC [83], which contains a promoterless firefly luciferase (Fluc) reporter gene and a renilla luciferase (Rluc) reference gene driven by the CaMV 35S promoter. Meanwhile, the CDS of *SlDML2* without the stop codon was amplified from tomato cDNA using the primers F (5′-ATGGAAACAGGCCAAGGCAG-3′) and R (5′-GGAGGCTACTCCTTTGTCTTC-3′) and then ligated into the pCambia2300-MCS-HA vector. The constructed plasmids were transformed into *Agrobacterium tumefaciens* strain GV3101. The *Agrobacterium* was grown at 28 °C for 24 h in Luria-Bertani (LB) medium supplemented with 50 μg mL^−1^ kanamycin, 50 μg mL^−1^ gentamycin, and 50 μg mL^−1^ rifampicin. Then, the cells were harvested and resuspended in the infiltration medium (10 mM MES, pH 5.6, 10 mM MgCl_2_, 100 μM acetosyringone) to a final OD_600nm_ of 0.3. The *Agrobacterium* harboring the reporter vector was then coinfiltrated into the *N. benthamiana* leaves with the *Agrobacterium* carrying the *SlDML2*-expressing vector (pCambia2300-*SlDML2*-HA) or the control vector (pCambia2300-HA) at 1:1 ratio. After incubation at 22 °C for 36 h, the agroinfiltrated leaves were collected and the activity of cytosol-synthesized Fluc was detected after spraying 1 mM luciferin and displayed by chemiluminescence with pseudo-color. The Fluc activity was also quantitatively analyzed using a dual-luciferase assay kit (Promega, E1910). The analysis was executed using the Ultra-Sensitive and Versatile Single Tube Luminometer (Promega, E5311) according to the manufacturer’s instructions. At least six measurements were contained for each assay.

### 5mC assay for tomato *ALKBH* promoters

The 5mC levels of promoters of tomato *ALKBHs* in the fruit of wild type at various ripening stages and the fruit of *Cnr* or *sldml2* mutant were analyzed on the base of tomato epigenome database (http://ted.bti.cornell.edu/epigenome/) or tomato DNA methylome database produced by Lang et al. [38].

The 5mC levels of *SlALKBH2* promoter in *N. benthamiana* were assessed as previously described with minor modifications [84]. In brief, genomic DNA was extracted from the agroinfiltrated *N. benthamiana* leaves, and 500 ng of purified DNA was treated with bisulfite to produce mutations from cytosine (C) to thymine (T) using EZ DNA methylation-gold kit (ZYMO Research, D5005). Then, 100 ng of mutated DNA was used as templates for PCR amplification with the primers F (5′-GTCAACTTAGATGATACGTAGAGACATTG-3′) and R (5′-CACAACCATGTACACACATGG-3′). The PCR products were cloned into pClone007 vector (TSINGKE, NMBV-007S), and at least 20 positive clones were detected by Sanger bisulfite sequencing. The sequencing results were analyzed on Kismeth (http://katahdin.mssm.edu/) to calculate the 5mC level based on the ratio of C-T mutations in each C site.

### Preparation of polyclonal antibodies

For SlALKBH2-specific antibody preparation, the full-length CDS of *SlALKBH2* was amplified from tomato cDNA using the primers F (5′-ATGGCCGGAGATTATAG-3′) and R (5′-TTATCTGCGACTTCTACGGC-3′) and inserted into the pET30a (+)-His-MCS vector (Merck KGaA). The resulting plasmid was transformed into *E. coli* BL 21 (DE3) competent cells for the expression of recombinant protein. The bacteria were cultured at 37 °C for overnight in LB medium containing 50 μg mL^−1^ kanamycin and then diluted 1:100 in 50 mL of fresh LB medium to continue culture until the OD_600nm_ reached at approximately 0.5. Then, isopropyl-1-thio-β-d-galactopyranoside (IPTG) was added to a final concentration of 1 mM to induce the expression of recombinant SlALKBH2 protein at 28 °C for 5 h. After induction, the bacterial cells were collected and dissolved in 5 mL 1× NTA binding buffer (50 mM Tris-HCl, pH 8.0, 300 mM NaCl, 10 mM imidazole). The cells were then broken by ultrasonication, followed by centrifugation at 4 °C, 10,000*g* for 10 min. The supernatant was mixed with 1 mL Ni-NTA His Bind Resin (Merck KGaA, 70666-4). Then, the mixture was incubated at 4 °C for 1 h. After washing three times with wash buffer (50 mM Tris-HCl, pH 8.0, 300 mM NaCl, and 20 mM imidazole), the bind resin was incubated with 1 mL elution buffer (50 mM Tris-HCl, pH 8.0, 300 mM NaCl, 250 mM imidazole) at 4 °C for 10 min to elute recombinant SlALKBH2 protein. The SlALKBH2 protein was further purified by 10% SDS-PAGE and used to immunize rabbits at Abmart (http://www.ab-mart.com.cn). SlALKBH2 polyclonal antibody was affinity-purified from antisera using the AminoLink Plus Coupling Resin (Thermo Scientific, 20501) according to the standard purification protocols.

### SlALKBH2 demethylation activity assay in vitro

The demethylation activity of SlALKBH2 protein in vitro was measured following the method of Jia et al. [25] with minor modifications. Briefly, 20 μg of recombinant SlALKBH2 protein prepared as described above and 1 nM of m^6^A-containing ssRNA, AUUGUC [m^6^A] CAGCAGC (synthesized at GenScript) were added to 100 μL of the reaction buffer (50 mM HEPES, pH 7.0, 100 mM KCl, 2 mM MgCl_2_, 2 mM l-ascorbic acid, 300 μM α-ketoglutarate, 150 μM (NH_4_)_2_Fe(SO_4_)_2_·6H_2_O, 300 U mL^−1^ RNase inhibitor). The reaction was carried out at room temperature for 6 h and then quenched by adding 5 mM EDTA followed by heating at 95 °C for 10 min. ssRNA was isolated from the reaction mix by using MiRNeasy mini kit (Qiagen, 217004) and digested by nuclease P1 (Wako, 145-08221). The digested substrates were analyzed on a HPLC system equipped with a 2489 UV/Vis detector (Waters, e2695), and the wave length for detection was set at 266 nm. Separation was performed at 22 °C on an Inertsil ods-3, C18, 5 μm analytical column (4.6 × 250 mm). The mobile phase consists of buffer A (25 mM NaH_2_PO_4_) and buffer B (100% acetonitrile). The analysis was performed at a 1 mL min^−1^ flow rate with the following buffer A/B gradient: 15 min 95%/5%, 5 min 90%/10%, and 1 min 100%/0%.

### SlALKBH2 demethylation activity assay in vivo

The demethylation activity assay of SlALKBH2 protein in vivo was performed with a transient expression system in *N. benthamiana*. Briefly, the *SlALKBH2* CDS without the stop codon was amplified from tomato cDNA by PCR using the primers F (5′-ATGGCCGGAGATTATAG-3′) and R (5′-TCTGCGACTTCTACGGC-3′) and inserted into the pCambia2300-MCS-HA vector. The resulting plasmid (pCambia2300-ALKBH2-HA) and the empty plasmid (pCambia2300-HA) were transformed into *A. tumefaciens* strain GV3101. The *Agrobacterium* was cultured at 28 °C for 24 h in LB medium. Then, the cells were harvested and resuspended in the infiltration medium (10 mM MES, pH 5.6, 10 mM MgCl_2_, 100 μM acetosyringone) to a final OD_600nm_ of 0.3 for infiltrating *N. benthamiana* leaves. After incubation at 22 °C for 36 h, *N. benthamiana* leaves were harvested and used for mRNA isolation and protein extraction. For in vivo demethylation activity assay, the abundance of mRNA m^6^A in *N. benthamiana* leaves with or without *SlALKBH2* expression was detected by LC-MS/MS assay as described above. The experiment was performed with three independent biological replicates.

### Subcellular localization

For subcellular localization analysis, the *SlALKBH2* CDS with the removal of stop codon was amplified from tomato cDNA using the primers F (5′-ATGGCCGGAGATTATAG-3′) and R (5′-TCTGCGACTTCTACGGC-3′) and then ligated into the pCambia2300-MCS-eGFP vector. The constructed plasmid was transformed into *A. tumefaciens* strain GV3101, which was subsequently used to infiltrate *N. benthamiana* leaves as described above for the expression of SlALKBH2-eGFP fusion protein. After incubation for 48 h, the mesophyll protoplasts were isolated from the agroinfiltrated leaves and observed under a Leica confocal microscope (Leica, DMI600CS). For an accurate localization, the RFP-HDEL fusion protein, a marker of the endoplasmic reticulum (ER), was co-expressed with the SlALKBH2-eGFP by using pBIN2-RFP-HDEL vector, which was kindly provided by Dr. Jinxin Lin (College of Biological Science and Technology, Beijing Forestry University). His-Asp-Glu-Leu (HDEL) is an ER retention signal peptide.

### Western blot analysis

For western blot analysis, proteins were separated by 10% SDS-PAGE and then electrotransferred to an Immobilon-P PVDF membrane (Millipore, IPVH00010) using a semi-dry transfer unit (Amersham, TE77). The membrane was blocked with 5% non-fat milk in PBST buffer for 2 h at room temperature. The immunoblotting was conducted by incubation with anti-ALKBH2 (1:10000), anti-HA (1:5000), anti-His (1:5000), or anti-ACTIN (1:5000) at room temperature for 2 h, followed by incubation with HRP-conjugated anti-rabbit IgG secondary antibody (1:10000) at room temperature for another 2 h. Immunoreactive bands were visualized by using the enhanced chemiluminescence detection kit as mentioned above. All commercial antibodies were purchased from Abmart (http://www.ab-mart.com.cn/).

### RNA immunoprecipitation

RIP was performed as previously described with minor modifications [29]. Briefly, tomato pericarps from wild-type fruit at 42 DPA were fixed with 1% formaldehyde on ice for 30 min under a vacuum. The fixation was terminated with 150 mM glycine for another 5 min. The fixed tissues (2 g) were ground and then homogenized in 2 mL of lysis buffer containing 50 mM HEPES, pH 7.5, 150 mM KCl, 2 mM EDTA, 0.5% NP-40 (*v*/*v*), 0.5 mM DTT, 2 mM EDTA, 300 U mL^−1^ RNase Inhibitor, and 1× cocktail protease inhibitor (Sigma, 04693132001). The homogenates were incubation at 4 °C for 1 h and then centrifuged at 15,000*g* for 30 min. Two hundred microliters of the supernatant was retained as the input sample. The remainder was subjected to immunoprecipitation (IP) with anti-ALKBH2 polyclonal antibody and rabbit IgG bound to Dynabeads Protein-A (Life Technologies, 10002A). Obtained IP samples and input samples were then heated at 55 °C for 10 min to reverse the RNA-protein cross-link. The immunoprecipitated RNA and input RNA were purified by phenol-chloroform extraction followed by ethanol precipitation. Equal amounts of RNA from each sample were reverse transcribed with an oligo (dT)_18_ primer using M-MLV reverse transcriptase (Takara, 2640A). Relative enrichment of individual gene was determined by quantitative RT-PCR using the primers listed in Additional file 11: Table S21. The experiment contained three biological replicates and each with three technical repeats.

### mRNA stability assay

The mRNA stability assay was performed with a transient expression system in *N. benthamiana*. In brief, the cDNA fragment of *SlDML2* composed of full-length CDS and 3′ UTR was amplified from tomato cDNAs. A mutated form of the amplified sequence with mutations from adenylate (A) to cytidine (C) in the potential m^6^A site located in the 3′ UTR of *SlDML2* cDNA was constructed using the QuikChange II XL site-directed mutagenesis kit (Agilent Technologies, 200518) following the manufacturer’s instructions. The two resulting fragments were then separately inserted into the pCambia2300-MCS-HA vector for the expression of intact or mutated *SlDML2* transcript. The constructing plasmids were introduced into *A. tumefaciens* strain GV3101. After cultivation, the *Agrobacterium* were coinfiltrated into the *N. benthamiana* leaves with the *Agrobacterium* carrying the *SlALKBH2*-expressing vector (pCambia2300-*SlALKBH2*-HA) or the control vector (pCambia2300-HA) at 1:1 ratio in a final OD_600nm_ of 0.6. After incubation for 24 h, leaf disks were taken from the infection parts of the *N. benthamiana* leaves and transferred onto the sterile water containing 10 μg mL^−1^ actinomycin D (Sigma, A4262). After 1 h of incubation, six leaf disks were collected and considered as time 0 controls, and subsequent samples were harvested every 3 h in triplicate. The expression level of *SlDML2* transcript was then determined by quantitative RT-PCR, and *N. benthamiana ACTIN* was used to normalize the expression values. All primers used for PCR amplifications were listed in Additional file 11: Table S22.

### CRISPR/Cas9 gene editing of *SlALKBH2*

CRISPR/Cas9 was performed as previously described [54] with minor modifications. In brief, CRISPR-P (version 2.0, http://crispr.hzau.edu.cn/CRISPR2/) was used to design the three specific sgRNAs containing different target sequences. sgRNA expression cassettes that driven by AtU6-1, AtU6-29, and AtU3b promoter, respectively, were amplified and cloned into the pYLCRISPR/Cas9Pubi-H binary vector [54] using the Golden Gate method. The resulting pYLCRISPR/Cas9Pubi-H-SlALKBH2 vector was transformed into *A. tumefaciens* strain GV3101. The *Agrobacterium* were grown in LB medium at 28 °C to a final OD_600_ of 0.5 and then used to agroinfiltrate the wild-type tomato Ailsa Craig according to the method of Fillatti et al. [55]. Mutation detections on transgenic lines were carried out by PCR amplifications using primers flanking the target sites, followed by sequencing with the internal primers (Additional file 11: Table S23). This mutation detection was also performed on potential targeted genes that predicted by CRISPR-P (version 2.0, http://crispr.hzau.edu.cn/CRISPR2/) to exclude the possibility of non-target mutations (Additional file 1: Figure S12).

## Additional files

Additional file 1: Supplementary Figures S1–S12 and supplementary text. (PDF 2428 kb)

Additional file 2: Supplementary Table S1. (XLSX 11 kb)

Additional file 3: Supplementary Tables S2–S4. (XLSX 2648 kb)

Additional file 4: Supplementary Table S5. (XLSX 10 kb)

Additional file 5: Supplementary Tables S6–S9. (XLSX 323 kb)

Additional file 6: Supplementary Tables S10–S11. (XLSX 2037 kb)

Additional file 7: Supplementary Tables S12–S15. (XLSX 113 kb)

Additional file 8: Supplementary Tables S16–S17. (XLSX 68 kb)

Additional file 9: Supplementary Table S18. (XLSX 12 kb)

Additional file 10: Supplementary Table S19. (XLSX 12 kb)

Additional file 11: Supplementary Tables S20–S23. (XLSX 17 kb)

Additional file 12: Review history. (DOCX 15 kb)

## Data Availability

The raw sequencing data and processed peaks data in m^6^A-seq have been deposited in the Gene Expression Omnibus database under the accession number GSE125306 [85] (https://www.ncbi.nlm.nih.gov/geo/query/acc.cgi?acc=GSE125306). All the other data generated in this study are included in the article and the additional files.
