# Supplementary material for: RNA methylomes reveal the m6A-mediated regulation of DNA demethylase gene SlDML2 in tomato fruit ripening
Source: Genome Biol. 2019 Aug 6;20:156. doi: 10.1186/s13059-019-1771-7 (PMC6683476; doi:10.1186/s13059-019-1771-7)
Supplement: Supplementary file 1 — Supplementary Figures S1–S12 and supplementary text. (PDF 2428 kb) [file 13059_2019_1771_MOESM1_ESM.pdf]

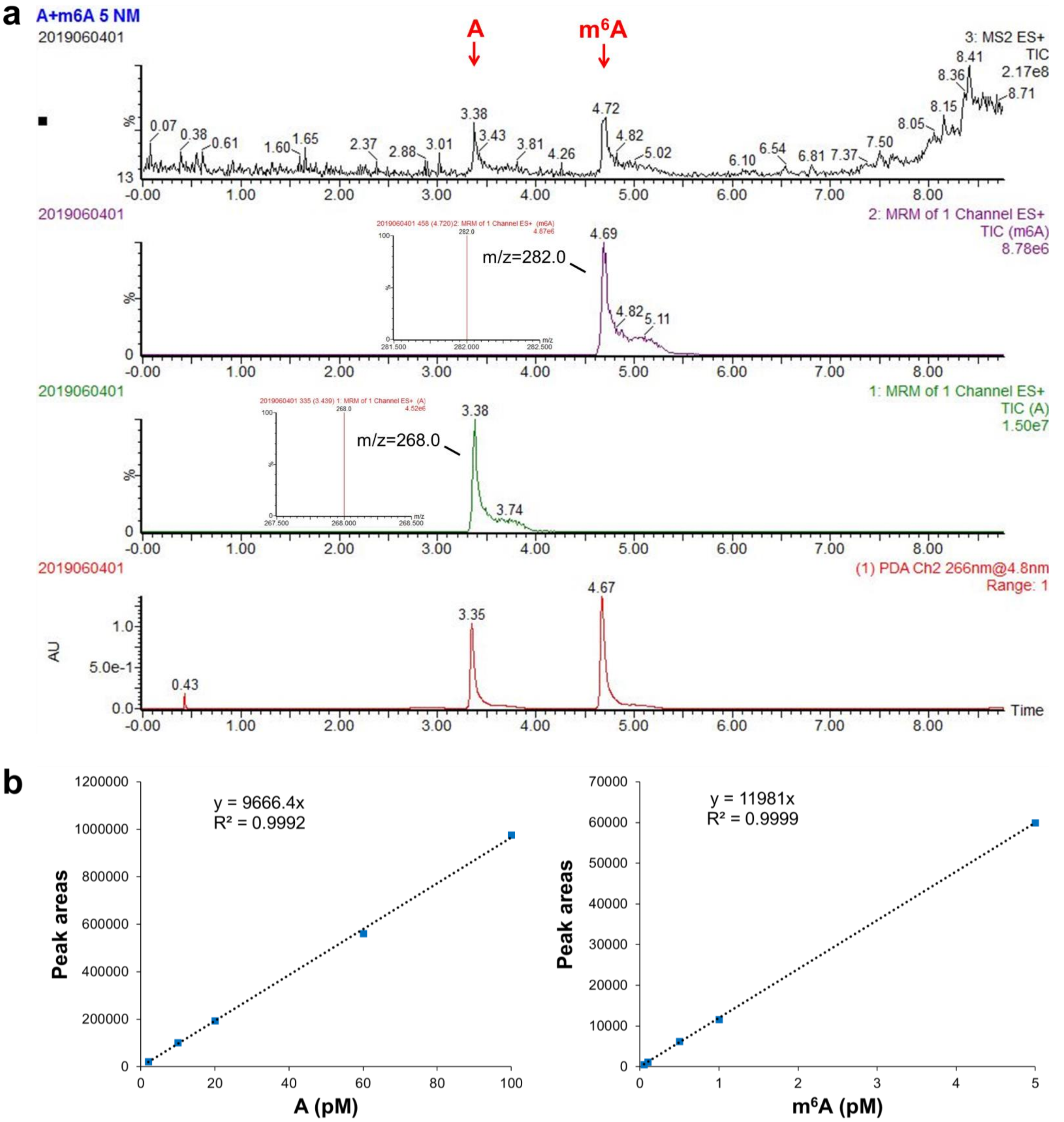

Fig S1. Representative LC-MS/MS chromatogram and the standard curves for A and m^{6}A. **a** The amount of A and m^{6}A was quantified by its integration area in the corresponding chromatogram. The m/z of A and m^{6}A is 268.0 and 282.0, respectively. **b** The A and m^{6}A standard curves obtained from five standard samples with different amounts of pure A and m^{6}A, respectively, by LC-MS/MS.

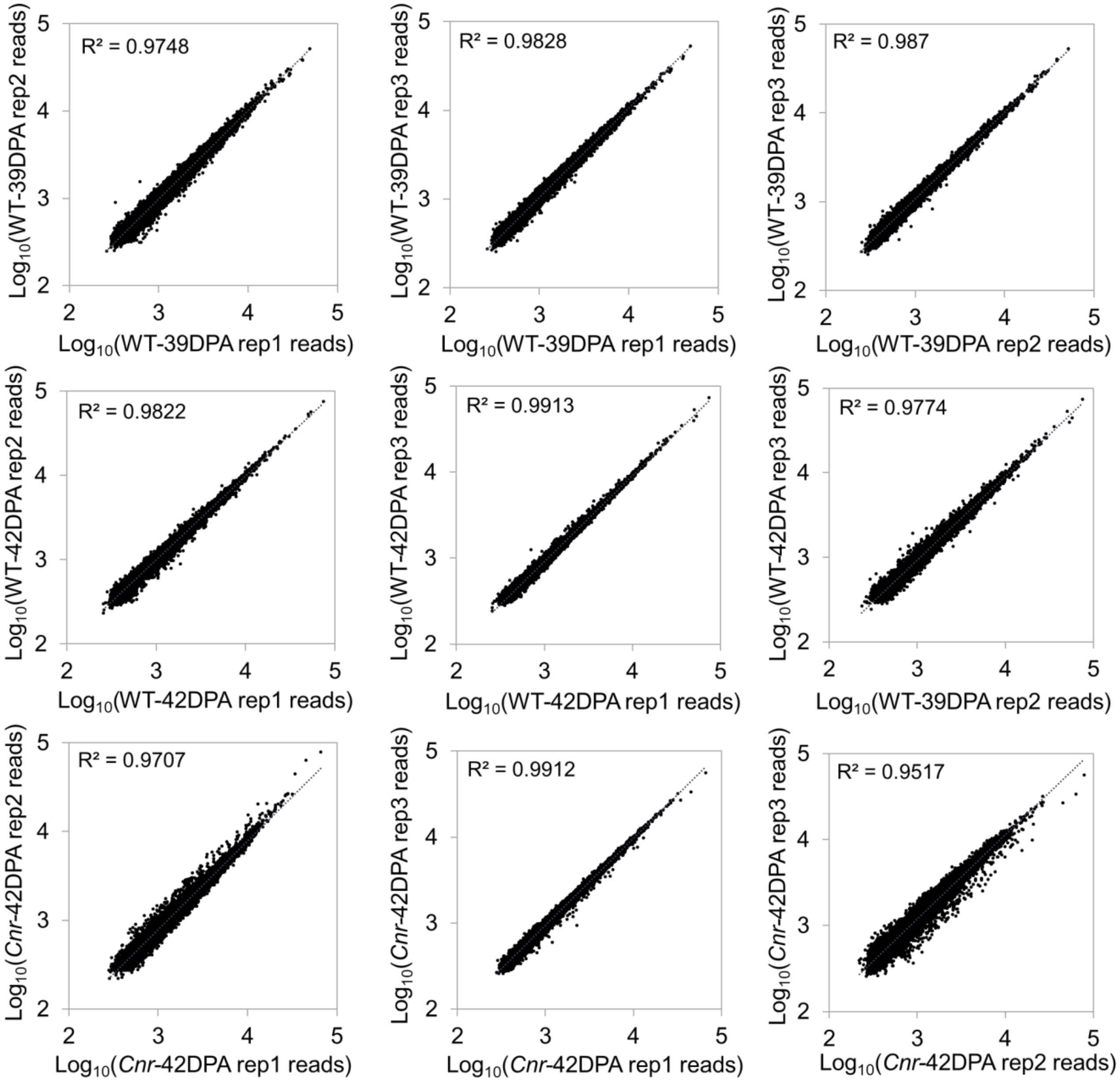

Fig S2. Pearson correlation analysis of reads of confident m^{6}A peaks between independent biological replicates in m^{6}A-seq. The m^{6}A-seq was performed on fruit of wild-type (WT) at 39 DPA and 42 DPA, in addition to fruit of *Cnr* mutant at 42 DPA. DPA, days post-anthesis; R, Pearson correlation coefficient; Rep, Replicate.

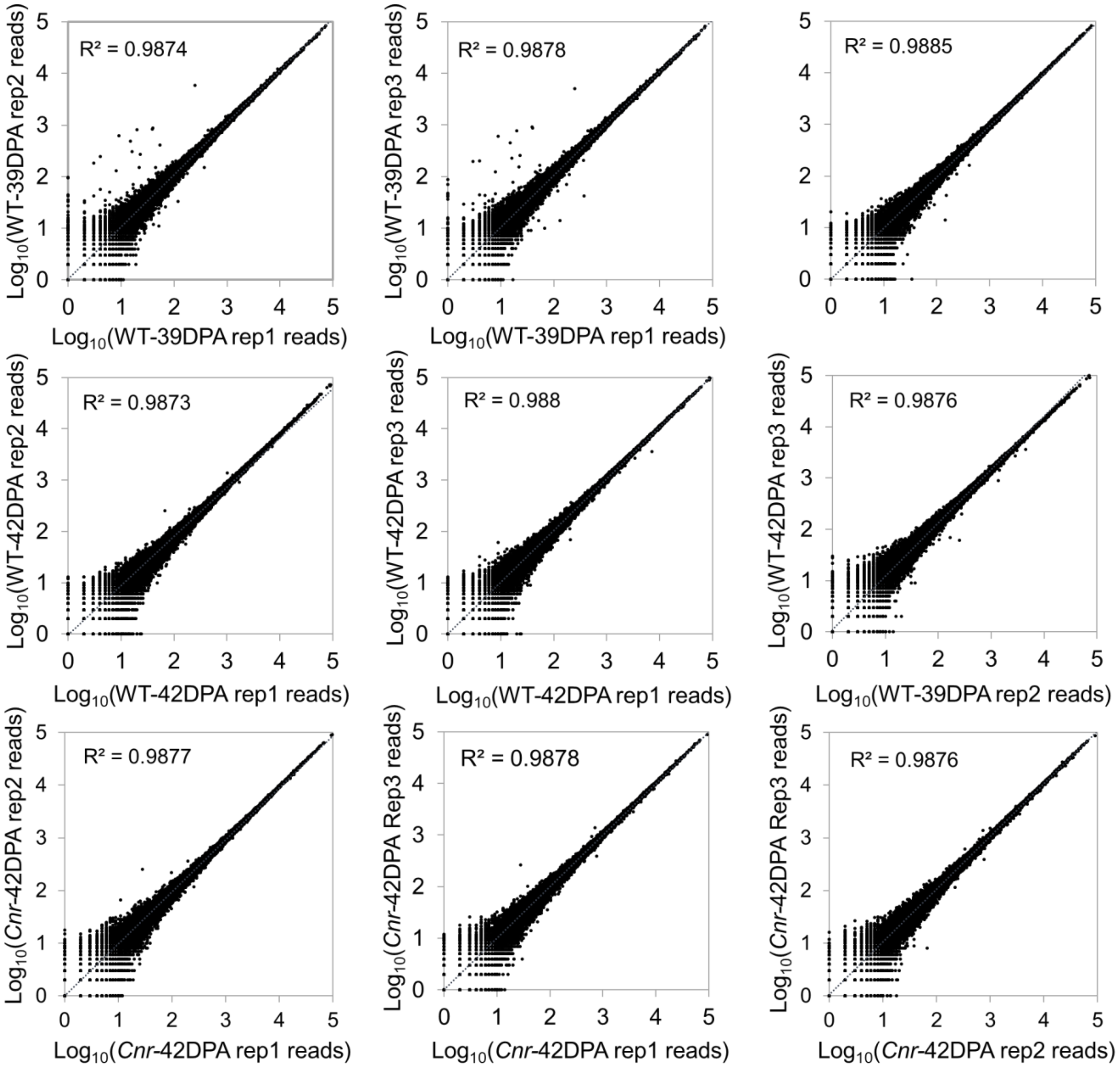

Fig S3. Pearson correlation analysis of reads of all transcripts between independent biological replicates in RNA-seq. The RNA-seq was performed on fruit of wild-type (WT) at 39 DPA and 42 DPA, in addition to fruit of *Cnr* mutant at 42 DPA. DPA, days post-anthesis; R, Pearson correlation coefficient; Rep, Replicate.

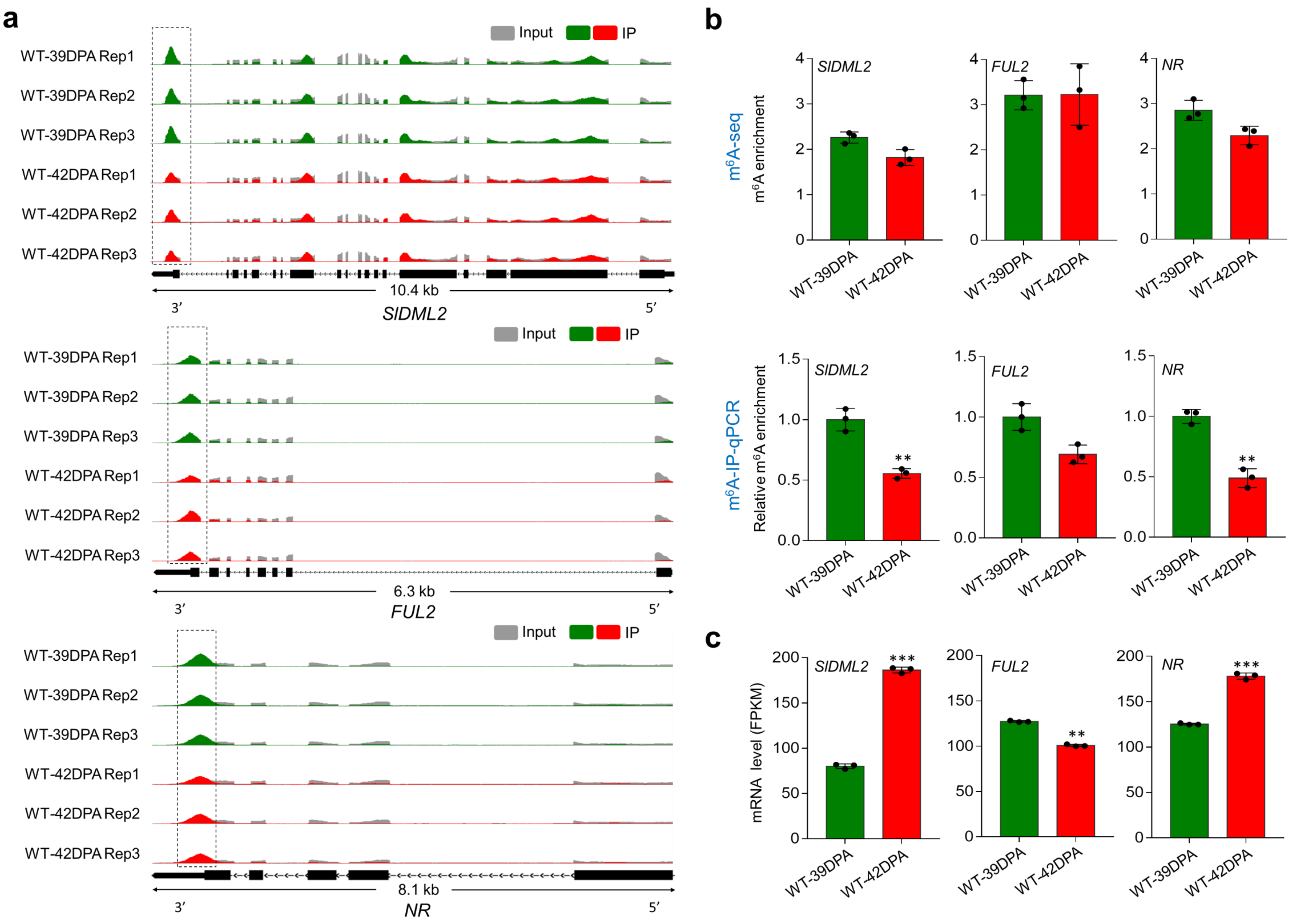

Fig S4. Changes in m^{6}A levels in transcripts of specific ripening-related genes during fruit ripening. **a** Integrated Genome Browser (IGB) tracks displaying m^{6}A-seq read distributions in *DEMETER-like DNA demethylase 2* (*SlDML2*), *fruitfull 2* (*FUL2*), and *never-ripe* (*NR*) transcripts in wild-type (WT) fruit at 39 DPA and 42 DPA. The black dot-line rectangles indicate the positions of m^{6}A peaks. **b** Validations of the m^{6}A enrichment by m^{6}A-immunoprecipitation (IP)-qPCR. **c** Gene expression level of *SlDML2, FUL2* and *NR* revealed by RNA-seq. In **b** and **c**, error bars represent the standard deviation of three independent experiments. Asterisks indicate significant differences (**P < 0.01, ***P < 0.001; Student's t-test).

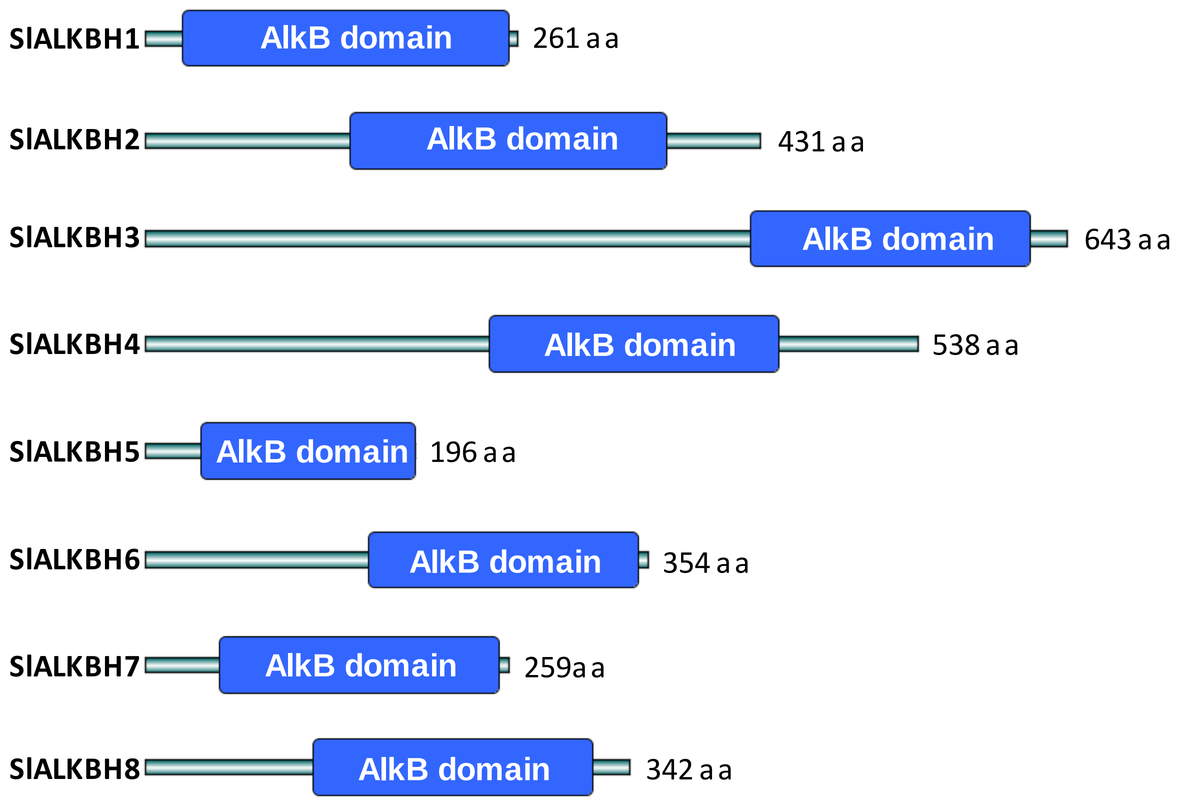

Fig S5. Identification of the highly conserved AlkB domain in tomato ALKBHs. These ALKBHs were named as SlALKBH1 to SlALKBH8 according to their location on tomato chromosomes. “aa” represents amino acid.

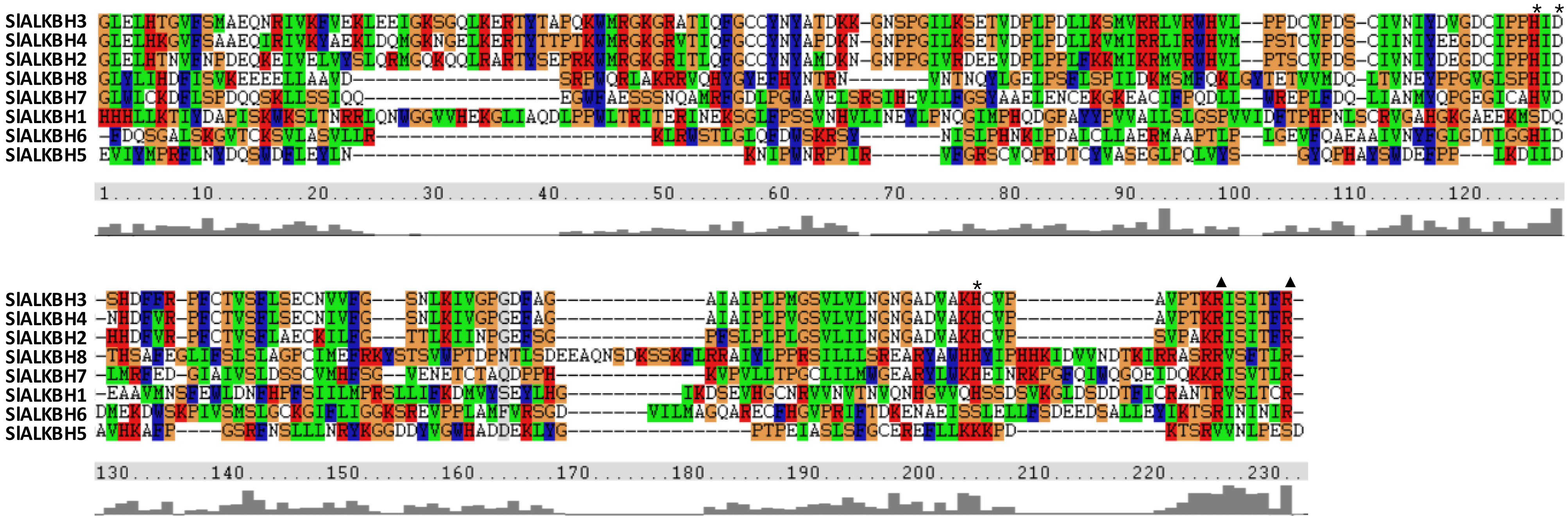

Fig S6. Protein sequence alignment of tomato ALKBHs using Clustal X. The amino acid sequences of AlkB domain in all tomato ALKBHs were used to generate the alignment. The conserved Fe(II) binding sites and α-ketoglutaramate binding sites were indicated by asterisks and triangles, respectively.

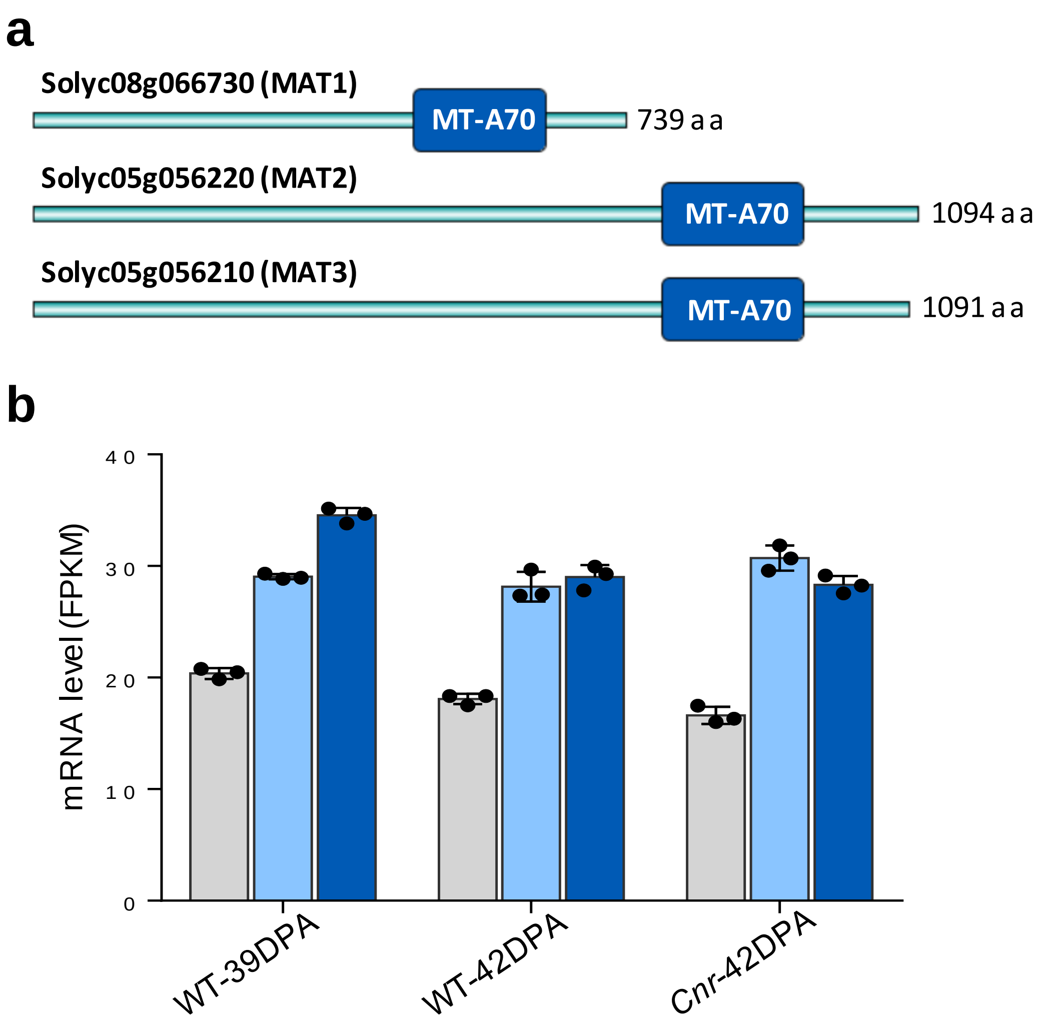

Fig S7. Expression analyses of potential tomato m^{6}A methyltransferase genes. **a** Identification of the highly conserved MT-A70 domain in three putative tomato m^{6}A methyltransferase (MAT1-3). Tomato m^{6}A methyltransferase candidates were identified by screening the SGN Tomato database using the *Arabidopsis* m^{6}A methyltransferase as a reference [28]. The MT-A70 domain was analyzed on pfam (http://pfam.xfam.org/). "aa" represents amino acid. **b** Transcript levels of the three putative tomato m^{6}A methyltransferase genes in wild-type (WT) fruit at 39 DPA and 42 DPA and *Cnr* fruit at 42 DPA. Error bars represent the standard deviation of three independent RNA-seq experiments. DPA, days post-anthesis; FPKM, fragments per kilobase of exon per million mapped fragments.

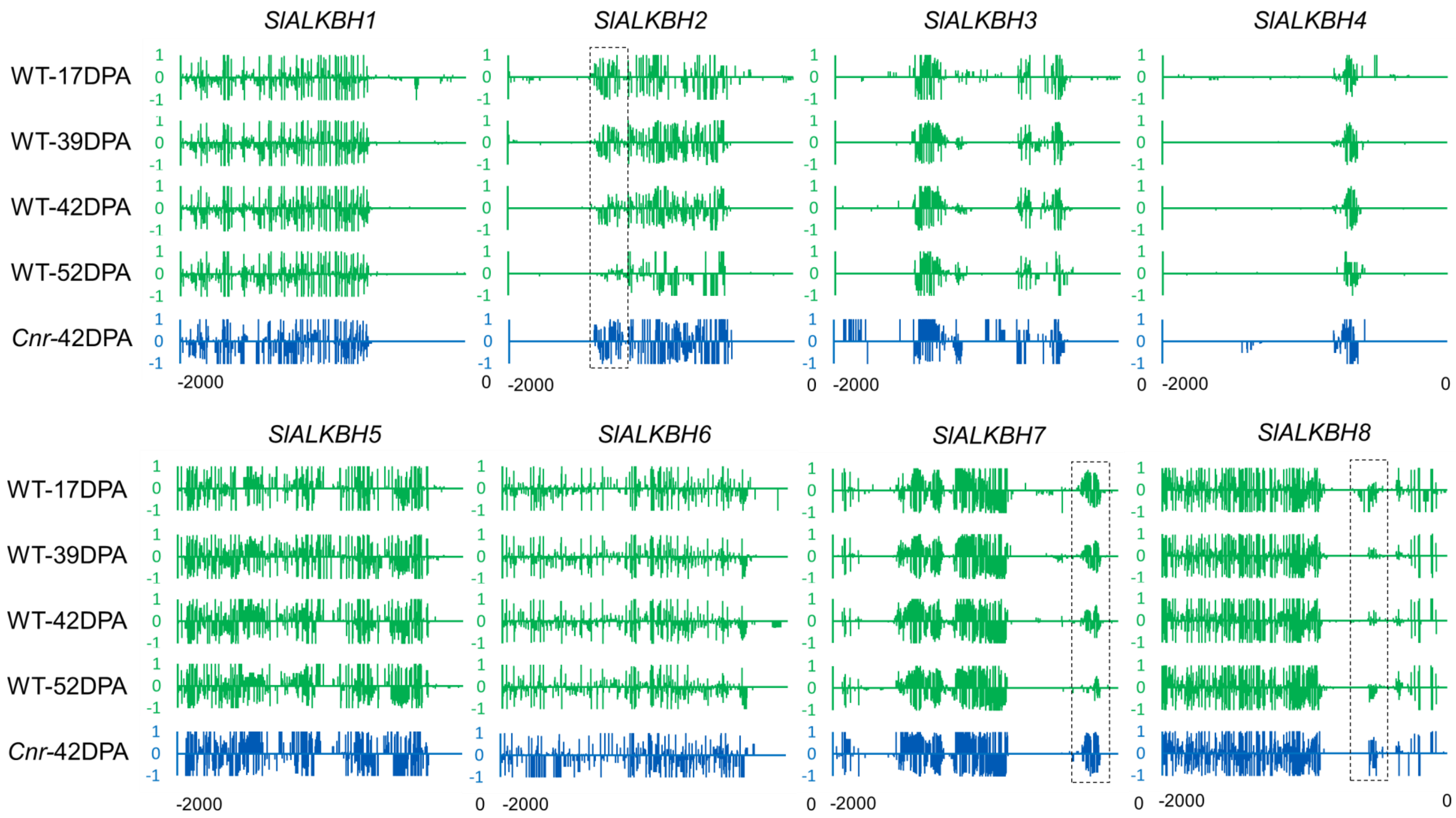

Fig S8. The 5mC levels in the promoter regions of tomato *ALKBHs*. These genes were named as *SlALKBH1* to *SlALKBH8* according to their location on tomato chromosomes. The 5mC levels in the 2000 bp upstream of the start codon in each gene were analyzed in fruit of wild-type (WT) and *Cnr* mutant using the Tomato Epigenome Database (http://ted.bti.cornell.edu/epigenome/). DPA, days post-anthesis. Each vertical bar represents a 5mC and the height of the bar indicates methylation level. The black dot-line rectangles indicate the positions of differentially methylated regions (DMRs).

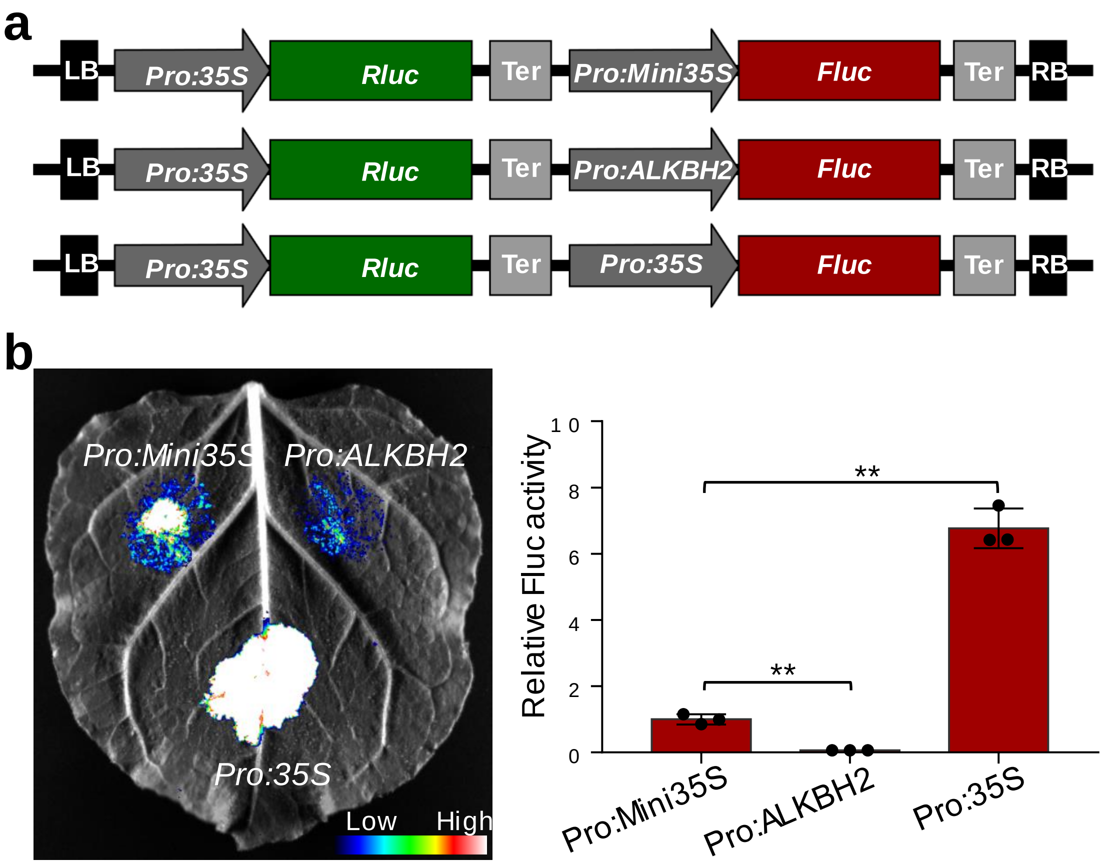

Fig S9. *SlALKBH2* promoter has the ability to activate firefly luciferase expression. **a** Schematic of dual-luciferase system used for promoter activity assay. The dual-luciferase reporter vector contains a firefly luciferase (Fluc) driven by mini35S, 35S, or *SlALKBH2* promoter and an internal control renilla luciferase (Rluc) driven by the 35S promoter. LB, left border; RB, right border; Ter, terminator. **b** Fluc activity assay in *Nicotiana benthamiana* leaves. The representative image from a total of six images is shown (left panel). The Fluc activity was normalized against the Rluc activity (right panel). Error bars represent the standard deviation of three independent experiments. Asterisks indicate significant differences (**$P < 0.01$; Student's t-test).

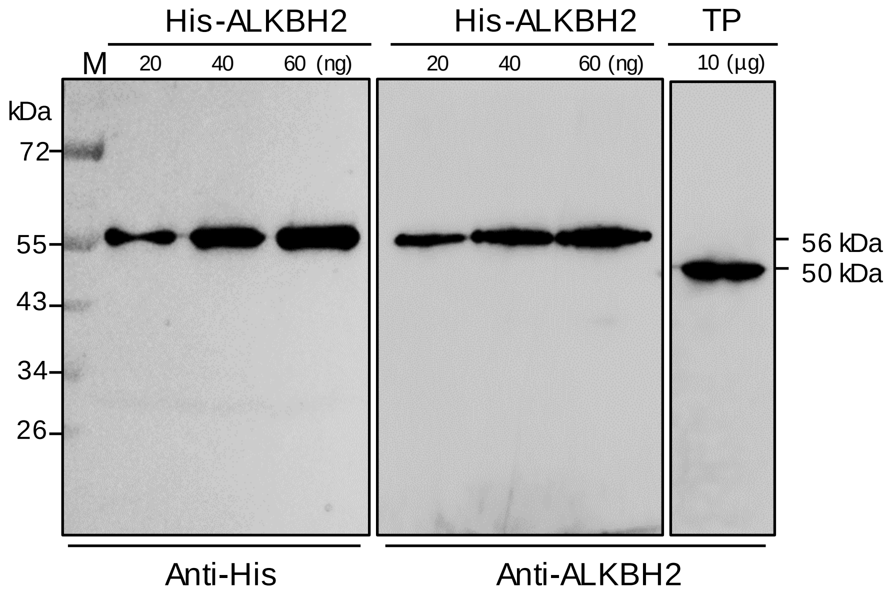

Fig S10. Immunoblot assay showing the specificity of anti-ALKBH2 antibody. Recombinant His-ALKBH2 protein expressed in *Escherichia coil* and total protein (TP) extracted from wild-type tomato fruit at 42 days post-anthesis were used for immunoblot assay with both anti-His and anti-ALKBH2 antibodies. The endogenous SlALKBH2 protein has a predicted molecular mass of ~50 kDa, and the recombinant His-ALKBH2 protein has a predicted molecular mass of ~56 kDa. M, protein maker. The experiment was repeated in three times and representative results are shown.

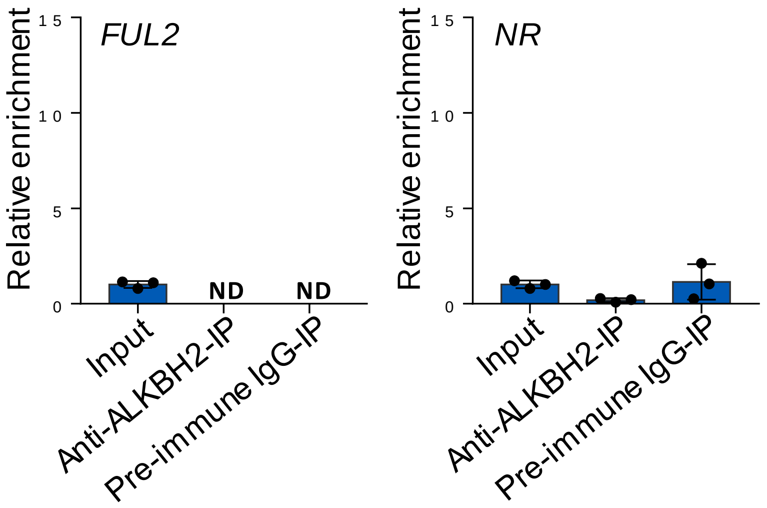

Fig S11. RNA Immunoprecipitation (RIP) assay showing SlALKBH2 protein do not bind *fruitfull 2* (*FUL2*) or *never-ripe* (*NR*) transcripts. For the RIP assay, the protein-RNA complexes were extracted from wild-type tomato fruit at 42 days post-anthesis and subjected to immunoprecipitation with anti-ALKBH2 polyclonal antibody or rabbit IgG (negative control). Error bars represent the standard deviation of three independent experiments. ND, not detected.

ITAG ID	Target site	Sequence	Off-score	Region	PCR primers	Internal primers	off-target ?
Solyc03g093510.2	1	GCTACTTTACCAACTA AGGATGG	0.174	Intron	PCR-F: GCTGAGGAAGAAAGGTTAATTTATG PCR-R: CTTTGAGGTCTTCTTATCAAAAGAAG	GGTGAAGCTTACTCTTGGATGAG	NO
Solyc12g098930.1	1	GGGAGTCAAACAACT CAAGAAAG	0.065	CDS	PCR-F: GTTAGAACGTTCTTATTGTTGCTTC PCR-R: GAAAACAACTGTAGCAACCATGAC	CTGTTGTTTTTCTGGTGTTTGATC	NO
Solyc04g016260.2	1	GCCACTGAACCACCT CATGACGG	0.025	CDS	PCR-F: GCGAAAGAAAGTGCTGATATTGTAG PCR-R: CTTTGAGGTCTTCTTATCAAAAGAAG	GACTGTCAATGTTGTGGCTTTG	NO
Solyc01g094000.2	2	TAATATCTAACCATGAC CGGCGG	0.109	CDS	PCR-F: GAAGACTGTTTTTAGTTTGCAG PCR-R: CAAACTCATTATTTGAATTACTCGC	GGAGAAATCCAATGAACAGAC	NO
Solyc05g054740.2	2	TCTTCTCTGACACTTC CCGGTGG	0.014	UTR	PCR-F: GCAGTTTATAGTTTTTCCGCC PCR-R: CGAACAACTATGTTTCATACGG	GTACCTGAGGAACAAGCATG	NO
Solyc01g098340.2	3	GATGACATCCATGCTA CTGATGG	0.019	CDS	PCR-F: GGAGCTCTGATCCAGATAATC PCR-R: CAAGGAGGACTACATGCAATTTG	GTTTCTGATTATGTTGTGGATG	NO
Solyc06g074010.2	3	GCTGACACCAATCTCA GGGAAAG	0.004	CDS	PCR-F: CTGAGCTGTTTCTCATGCCTAG PCR-R: GCTCTTACCCCAATGGACAG	GTCAAATCATGTCATAGGTGGC	NO

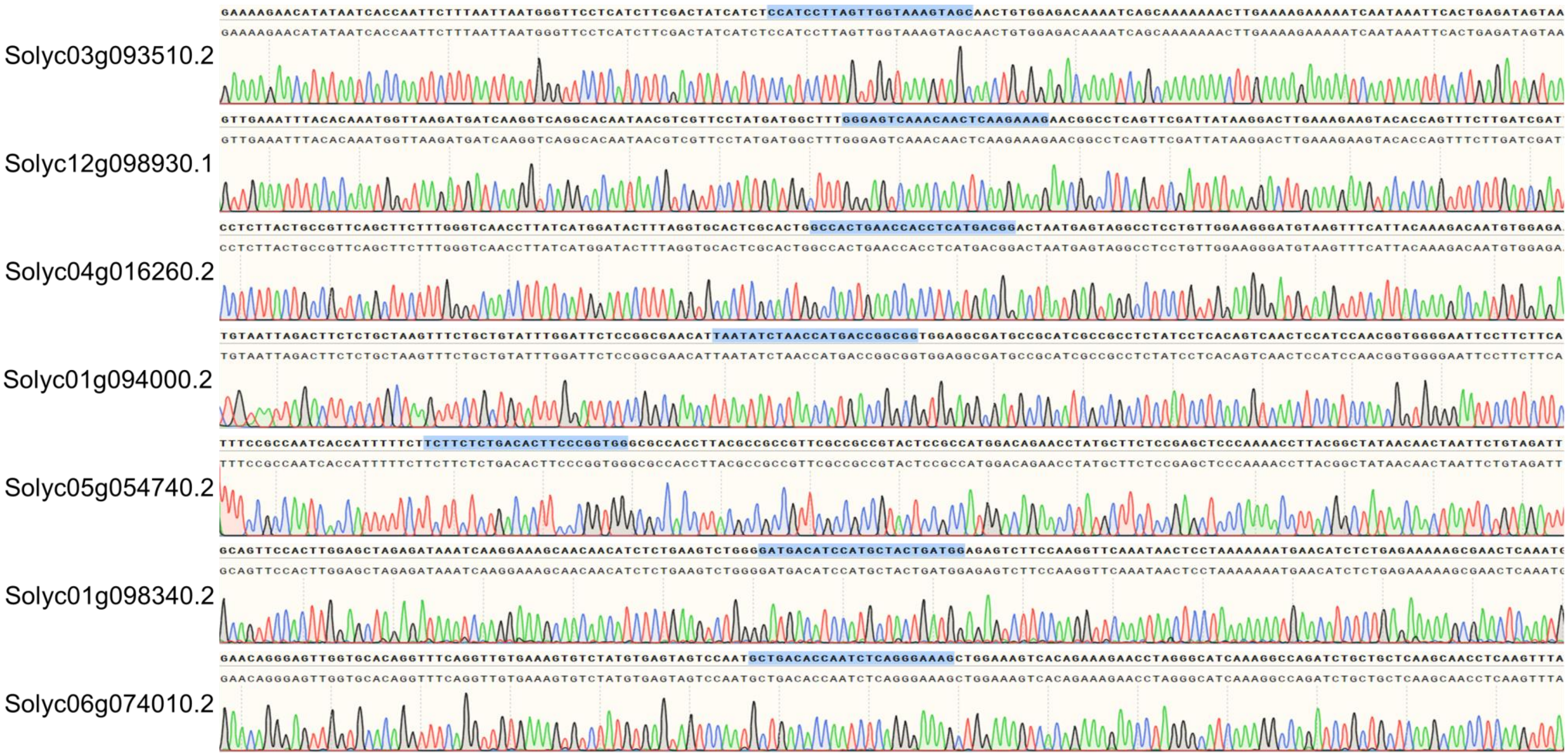

Fig S12. Off-target analysis in *slalkbh2-23*, *slalkbh2-25* and *slalkbh2-28* mutants. Potential targets and off-target genes were predicted by CRISPR-P (version 2.0, http://crispr.hzau.edu.cn/CRISPR2/). Red letters indicate the protospacer adjacent motif (PAM). The potential off-target genes were genotyped through direct sequencing of PCR products from genomic DNA flanking the potential target sites. Sequencing chromatograms of 150-bp nucleotides around the potential targets were shown. Potential targets were indicated with blue background.

Supplementary text. Protein sequences of tomato ALKBHs.

>SlALKBH1_Solyc01g057570.2
MEMSKTLNEFRVGSVPTVFYIPDFVTESEHHHLLKTIYDAPISKWKSLTNRRLQNWGGVVHEKGLIAQDLPPWLTRITERINEKSGLFPSSVNHV
LINEYLPNQGIMPHQDGPAYYPVVAILSLGSPVVIDFTPHPNLSCRVGAHGKGAEEKMSDQEAAVMNSFEWLDNFHPFSIILMPRSLLIFKDMVY
SEYLHGIKDSEVHGCNRVVNVTNVQNHGVVQHSSDSVKGLDSDDTFICRANTRVSLTCRVVTKVHKSIFKF

>SlALKBH2_Solyc01g104130.2
MAGDYSRWSPEPRDFKAMLKKLSHEQLLQVLSEGLCSYCEEVIESRVNNLNFIQKSSSDDAISPKRKLNADAYTPFPQVGDSTNSGRSKRFVS
ALSKPVINKQPASDLSNFEIKLNDGPSRSVVGDDLVEDQKEFIRFSQVGRRKDFVHYENVNEKQINVLKGLELHTNVFNPDEQKEIVELVYSLQR
MGQKQQLRARTYSEPRKWMRGKGRITLQFGCCYNYAMDKNGNPPGIVRDEEVDPLPPLFKKMIKRMVRWHVLPTSCVPDSCIVNIYDEGDCI
PPHIDHHDFVRPFCTVSFLAECKILFGTTLKIINPGEFSGPFSLPLPLGSVLILNGNGADVAKHCVPSVPAKRISITFRKMDVSKLPYRYTPDPELV
GIERLIPSPSLDSSRNRYHGKIDKLPNSENKVFSNEDDFPPLGKSTSSSRRSRR

>SlALKBH3_Solyc02g062180.2
MLDHHHRSSTTDRFLLDYNADELRIAGEFLSNWFPFLSRDFCSSCTHTLSHRIRSLGRREAVGDAEQLKQQENFVVLTPELPDSNACNGNHDN
CYGNSLGSWQDCAVLNGSADTNSLGSWKDGAVVQEPFDDALTCRNKSNSYVGGRSLGSLKGYADLNDTADTNSLGSWKDGADVQEPFDEA
LAPRNKSDNNVGGAVHPLGSWKDYADLNDTADTNSLGSWKDGADVQEPFDEALAHRNKSDSYIGVADRPIGSWKDCADLNDNVDTNSLGSW
KDSADVQEPFEGALTPRIKPDNYLGGAARPVKEASRSKTFRSSRPTAIWRMKMPLADELDAVEISESSICSSQLRNGNGMNKEETSVQGAKPK
MELSMEQREHIRFCNVKRKKDFICLERVNGKIVNILDGLELHTGVFSMAEQNRIVKFVEKLEEIGKSGQLKERTYTAPQKWMRGKGRATIQFGC
CYNYATDKKGNSPGILKSETVDPLPDLLKSMVRRLVRWHVLPPDCVPDSCIVNIYDVGDCIPPHIDSHDFFRPFCTVSFLSECNVVFGSNLKIVG
PGDFAGAIAIPLPMGSVLVLNGNGADVAKHCVPAVPTKRISITFRRMNESRRPIGCAPEQDLLGLQPISHEADRYEKSKTYKPWHSK

>SlALKBH4_Solyc02g083960.2
MSDHQRTRKDDPFLLNYNSDELRIASEFLSNWLPFLSRDLCRSCTHTLSDRIRSLNSKVSGNAECLKQPKNVSVLTPERCDSDGCNGSQENC
DNNSLGSWNDYGDLNDNADTNSLGSWKDGAGGEPFEELSVDRNSSDGCIDGADSQSQSVGEASTSEAFKSSLPVTTPKVKMSWADMAQED
ELQAEEVSESIALRSQVNGVAEEEITNPESKQKTELSREKREYIRFCNVKRKKDFICLERVRGKIVNILDGLELHKGVFSAAEQIRIVKYAEKLDQ
MGKNGELKERTYTTPTKWMRGKGRVTIQFGCCYNYAPDKNGNPPGILKSETVDPLPDLLKVMIRRLIRWHVMPSTCVPDSCIINIYEEGDCIPP
HIDNHDFVRPFCTVSFLSECNIVFGSNLKIVGPGEFAGAIAIPLPVGSVLVLNGNGADVAKHCVPAVPTKRISITFRRMDESKRPTGYVPEHDLQG
LQPLSYESDSQKKSSSSRPRFSARKQSVRQEEESRERVKMPMRRHSEPRYPGRYRGGPANRQWYGANTEN

>SlALKBH5_Solyc04g015080.2
MATVTKLNLKEETGDCHRLEAEQSEAPAEISDSRTKRVVELGNGSEVIYMPRFLNYDQSWDFLEYLNKNIPWNRPTIRVFGRSCVQPRDTCYV
ASEGLPQLVYSGYQPHAYSWDEFPPLKDILDAVHKAFPGSRFNSLLLNRYKGGDDYVGWHADDEKLYGPTPEIASLSFGCEREFLLKKKPDKT
SRVVNLPESD

>SlALKBH6_Solyc04g045590.2
MYGPTEDAERTAFRMAEKKYKLYYDNTRKKKQPRPVDLSDVTDFKSISEAYHRNAELPSGIFPIHCDLHTPIFCLESHPGFYFIPGALPVEEQCR
WIKESLTSFPQPPNRTNHNAIYGPLQDLFAAAKDNKVLIQEEQYCGTNNSEVEIIENDINVPTWNFFDQSGALSKGVTCKSVLASVLLRKLRWST
LGLQFDWSKRSYNISLPHNKIPDALCLLAERMAAPTLPLGEVFQAEAAIVNYFGLGDTLGGHLDDMEKDWSKPIVSMSLGCKGIFLIGGKSREV
PPLAMFVRSGDVILMAGQARECFHGVPRIFTDKENAEISSLELLFSDEEDSALLEYIKTSRININIRQVF

>SlALKBH7_Solyc09g074920.2
MDELKMILTEAFGDSSNSEGEEEEQFLHVHSVENKVNGKALIRSVFGETHNWERISEIDGLWLCKDFLSPDQQSKLLSSIQQEGWFAESSSNQ
AMRFGDLPGWAVELSRSIHEVILFGSYAAELENCEKGKEACIFPQDLLWREPLFDQLIANMYQPGEGICAHVDLMRFEDGIAIVSLDSSCVMHFS
GVENETCTAQDPPHKVPVLLTPGCLILMWGEARYLWKHEINRKPGFQIWQGQEIDQKKRISVTLRKLGRTD

>SlALKBH8_Solyc12g096230.1
MGLPRFKRPVGTSEPSSNLYVANCGPAVGLTLDTIEAVFGAYGQVKGVHLADESGTRVIVSYHEEKSAESALTALNRRACPELGGRSLHIQYSV
PSVCQVAVDDSIEVSMESSELDIPGLYLIHDFISVKEEEELLAAVDSRPWQRLAKRRVQHYGYEFHYNTRNVNTNQYLGELPSFLSPILDKMSMF
QKLGYTETVVMDQLTVNEYPPGVGLSPHIDTHSAFEGLIFSLSLAGPCIMEFRKYSTSVWPTDPNTLSDEEAQNSDKSSKFLRRAIYLPPRSILLL
SREARYAWHHYIPHHKIDVVNDTKIRRASRRVSFTLRKVRKGPCECEFPEYCDSQKC
