# Supplementary material for: RNA methylomes reveal the m6A-mediated regulation of DNA demethylase gene SlDML2 in tomato fruit ripening
Source: Genome Biol. 2019 Aug 6;20:156. doi: 10.1186/s13059-019-1771-7 (PMC6683476; doi:10.1186/s13059-019-1771-7)
Supplement: Supplementary file 12 — Review history. (DOCX 15 kb) [file 13059_2019_1771_MOESM12_ESM.docx]

Review History

**First round of review**

**Reviewer 1**

**Are you able to assess all statistics in the manuscript, including the appropriateness of statistical tests used?**

No, I do not feel adequately qualified to assess the statistics.

**Comments to author:**

Zhou et al found RNA modification m6A plays function in tomato fruit ripening, and identified a tomato m6A demethylase and additionally revealed the link between DNA 5mC and RNA m6A modification during fruit ripening. It is a very interesting paper.

1)	The authors made a model that during fruit ripening, DNA demethylase SlDML2 regulates the transcription of m6A demethylase SIALKBH2, which removes m6A from SIDML2 mRNA, therefor regulating its stability, which forms a feedback regulation loop. From the results, both of the changed total m6A levels and the changed expression levels of SIALKBH2 between WT-39DPA vs WT-42DPA and WT-42DPA vs cnr-42DPA are similar, however, the m6A-seq analysis found only 240 transcripts with increased m6A peaks in WT-39DPA but 1107 transcripts with higher m6A levels in cnr-42DPA compared to WT-42DPA. These results are completely inconsistent with the results from dot-blot and expression levels of SIALKBH2.

2)	The authors used a spontaneous epimutant Cnr line to seek the mechanism. They did not confirm whether this mutant is a single mutant line. Although the expression levels of many genes including SIALKBH2 are varied, it is hard to clarify it is a clue or consequence. Thus the authors should show whether the phenotype is recovered when complement of SIALKBH2 into cnr.

3)	Although the author showed a phenotype of RNAi KD SIALKBH2. The authors should show statistic data ( how many tomato have the phenotype (n out of m) in RNAi KD SIALKBH2 and control, repectively. Whether it is significant).

4)	In figure 8C, the authors should show the original HPLC chromatogram, not the bar figures. Importantly, the ratio of A to m6A in the sequence used is 3:1, however, from the non enzyme data, the amount of A is 1.5-fold of m6A. Clearly this quality of this assay is not acceptable. Maybe the RNA digestion is uncompleted. Thus I am not confident of the in vitro demethylation activity.

5)	Dot-blot is not a quantitative method. It is surprised the error bar is so small. I suggested the author use LC-MS/MS to quantitatively measure m6A levels.

6) whether total m6A level is increased in RNAi KD SIALKBH2?

===

Please consider the following questions:

- Are the methods appropriate to the aims of the study, are they well described, and are necessary controls included? If not, please specify what is required.

No, SlALKBH2 demethylation activity assay in vivo, m6A-IP-qPCR, protein purification

- Are the conclusions adequately supported by the data shown? If not, please explain

Yes, but need some additional assay to support, see comments.

- Are sufficient details provided to allow replication and comparison with related analyses that may have been performed? If not, please specify what is required.

Yes, but need some additional assay to support, see comments.

- Does the work represent a significant advance over previously published studies?

Yes

- Is the paper of broad interest to others in the field, or of outstanding interest to a broad audience of biologists?

yes

**Reviewer 2**

**Are you able to assess all statistics in the manuscript, including the appropriateness of statistical tests used?**

No, I do not feel adequately qualified to assess the statistics.

**Comments to author:**

The manuscript by Zhou et al presents a relatively comprehensive and convincing study concerning the role of the demethylase gene SlDML2 in tomato fruit ripening. The experiments appear to be well planned, executed and described.

One aspect that is problematic is the use of VIGS to prove ripening phenotypes. This MUST be repeated via stable transformation. Many, many VIGS experiments reveal ripening phenotypes and this in itself would not appear to be solid proof. Given that stable transformation of tomato is facile I recommend that this approach is taken since it is considerably more reliable. Whilst I expect the authors will reveal a similar result it is needed to be demonstrated that they indeed do so!

There are a number of typos which need correcting "Since cnr" alpha-ketoglutaramate for example. This needs a careful review.

**Authors Response**

**Point-by-point responses to the reviewers’ comments:**

We would like to thank the reviewers for their constructive and positive feedback. Based on the reviewers’ suggestions we have carried out additional experiments and substantially revised the manuscript. We believe that our manuscript has been improved by incorporating and implementing the reviewers’ comments. Details of the changes made during the revision are highlighted in the manuscript.

Response to the comments of Reviewer #1:

Zhou et al found RNA modification m6A plays function in tomato fruit ripening, and identified a tomato m6A demethylase and additionally revealed the link between DNA 5mC and RNA m6A modification during fruit ripening. It is a very interesting paper.

Question 1: The authors made a model that during fruit ripening, DNA demethylase SlDML2 regulates the transcription of m6A demethylase SlALKBH2, which removes m6A from SlDML2 mRNA, therefore regulating its stability, which forms a feedback regulation loop. From the results, both of the changed total m6A levels and the changed expression levels of SlALKBH2 between WT-39DPA vs WT-42DPA and WT-42DPA vs Cnr-42DPA are similar, however, the m6A-seq analysis found only 240 transcripts with increased m6A peaks in WT-39DPA but 1107 transcripts with higher m6A levels in Cnr-42DPA compared to WT-42DPA. These results are completely inconsistent with the results from dot-blot and expression levels of SlALKBH2.

*Response: We thank the reviewer for pointing this out. We have reanalyzed the total m6A levels using LC-MS/MS instead of dot-blot assay in the wild-type and Cnr mutant. The result indicated that the changed total m6A levels between WT-39DPA vs WT-42DPA is lower than those between Cnr-42DPA vs WT-42DPA (Fig. 1c; the ratio of WT-39DPA vs WT-42DPA is 1.16, while the ratio of Cnr-42DPA vs WT-42DPA is 1.54). This is consistent with the observation that only 240 transcripts were detected with increased m6A peaks in WT-39DPA but 1107 transcripts with higher m6A levels in Cnr-42DPA compared to WT-42DPA.*

*In our study, the changed expression levels of SlALKBH2 between WT-39DPA vs WT-42DPA and WT-42DPA vs Cnr-42DPA are similar. This suggests that the regulation of m6A is complicated and other factors in addition to SlALKBH2 might play roles in this process. Alternatively, SlALKBH2 could be regulated by translational or post-translational mechanisms. A sentence “Notably, the regulation of m6A is complicated and other factors in addition to SlALKBH2 might play roles in this process.” has been added in page 19 (highlighted) for discussion.*

Question 2: The authors used a spontaneous epimutant Cnr line to seek the mechanism. They did not confirm whether this mutant is a single mutant line. Although the expression levels of many genes including SlALKBH2 are varied, it is hard to clarify it is a clue or consequence. Thus the authors should show whether the phenotype is recovered when complement of SlALKBH2 into Cnr.

*Response: We thank the reviewer for this comment. The Cnr mutant has been previously characterized, using positional cloning, to harbor a naturally occurring epigenetic mutation in a gene encoding an SBP-box transcription factor (Manning et al., 2006. Nature Genetics, 38: 948-952). Whole-genome bisulfite sequencing to profile the DNA methylome demonstrated that Cnr mutant fruits exhibit genome-wide DNA hypermethylation (Zhong et al., 2013. Nature Genetics, 31: 154-159). Furthermore, DNA methylation-related genes such as CHROMOMETHYLASE3 (SlCMT3) and DOMAINS REARRANGED METHYLTRANSFERASE7 (DRM7) were shown to be essential for sustaining Cnr epi-allele and maintenance of Cnr phenotype in tomato (Chen et al., 2015. Scientific Reports, 5: 9192), confirming that Cnr phenotype is caused by DNA methylation.*

*In our study, we used Cnr mutant to seek the correlation between DNA methylation and mRNA m6A. We found that gene expression of SlALKBH2 declined dramatically in the Cnr mutant fruits (Fig. 6). Further analyses showed that SlALKBH2 promoter contains an obvious differentially methylated region (DMR), which remains hypermethylated in the Cnr mutant, but becomes demethylated during fruit ripening (Fig. 7). Moreover, demethylation of SlALKBH2 by expression of a DNA demethylase gene SlDML2 increased its transcript level (Fig. 7). These findings indicate that SlALKBH2 transcription is indeed regulated by DNA methylation. A sentence “The Cnr mutant has been previously characterized, using positional cloning, to harbor a naturally occurring epigenetic mutation in a gene encoding an SBP-box transcription factor [35].” has been added in page 6 (highlighted) to describe the background of Cnr mutant.*

Question 3: Although the author showed a phenotype of RNAi KD SIALKBH2. The authors should show statistic data (how many tomato have the phenotype (n out of m) in RNAi KD SlALKBH2 and control, respectively. Whether it is significant).

*Response: We have generated stable loss-of-function slalkbh2 mutants using the CRISPR/Cas9 gene-editing system to substitute VIGS experiment. Phenotype observation indicates that ripening of fruit from slalkbh2 mutants is delayed, which is similar to that observed in the VIGS experiment. The results and methods are described in page 17-19 and page 38-39 (highlighted), respectively. In addition, Fig. 10 has been revised.*

Question 4: In figure 8C, the authors should show the original HPLC chromatogram, not the bar figures. Importantly, the ratio of A to m6A in the sequence used is 3:1, however, from the non enzyme data, the amount of A is 1.5-fold of m6A. Clearly this quality of this assay is not acceptable. Maybe the RNA digestion is uncompleted. Thus I am not confident of the in vitro demethylation activity.

*Response: One of the original HPLC chromatogram is shown Fig. 8c in the revised manuscript. Based on your comments, we have carried out additional experiments. Similar results were obtained, and the amount of A, which is determined by peak area, is still 1.5-2.0 folds of m6A in non-enzyme data. Previous study also showed the difference between the ratio of A to m6A in the sequence and the detected amount of A to m6A by HPLC. For example, Duan et al. (2017) showed that the detected amount of A is around 0.5-fold of m6A, whereas the ratio of A to m6A in the sequence used is 6:1 (Duan et al., 2017. Plant cell, 29: 2995-3011; shown in Fig. 2B). We analyzed the peak area of pure A and m6A by LC-MS/MS, and found that the ratio of A to equal m6A is 0.8 (as shown in the standard curve in Additional file 1: Figure S1). We suppose that the ratio of nucleotide peak area is influenced by multiple factors, such as the characteristics of nucleotides, the reaction condition or the HPLC type.*

Question 5: Dot-blot is not a quantitative method. It is surprised the error bar is so small. I suggested the author use LC-MS/MS to quantitatively measure m6A levels.

*Response: According to your suggestion, we have used LC-MS/MS instead of dot-blot assay in the revised manuscript to quantitatively measure m6A levels. The results are described in page 7, 15, and 18 (highlighted) and the methods are shown in page 24-25 (highlighted). In addition, Fig. 1c, Fig. 8d, and Fig. 10 have been revised.*

Question 6: whether total m6A level is increased in RNAi KD SlALKBH2?

*Response: We have analyzed the total m6A levels in fruit of the slalkbh2-23, slalkbh2-25 and slalkbh2-28 mutants by LC-MS/MS in the revised manuscript, and found that the total m6A levels increased significantly in all three mutants compared with the wild-type. The results are added in page 18 (highlighted) and shown in Fig. 10c.*

Question 7: Please consider the following questions:

- Are the methods appropriate to the aims of the study, are they well described, and are necessary controls included? If not, please specify what is required.

No, SlALKBH2 demethylation activity assay in vivo, m6A-IP-qPCR, protein purification.

*Response: We have revised the Methods, including SlALKBH2 demethylation activity assay in vivo (page 35; highlighted), m6A-IP-qPCR (page 28-29; highlighted), and protein purification (page 33-34; highlighted).*

Response to the comments of Reviewer #2:

The manuscript by Zhou et al presents a relatively comprehensive and convincing study concerning the role of the demethylase gene SlDML2 in tomato fruit ripening. The experiments appear to be well planned, executed and described.

Question 1: One aspect that is problematic is the use of VIGS to prove ripening phenotypes. This MUST be repeated via stable transformation. Many, many VIGS experiments reveal ripening phenotypes and this in itself would not appear to be solid proof. Given that stable transformation of tomato is facile I recommend that this approach is taken since it is considerably more reliable. Whilst I expect the authors will reveal a similar result it is needed to be demonstrated that they indeed do so!

*Response: Thanks for your comments. We have been devoted to the generation of stable loss-of-function slalkbh2 mutants using the CRISPR/Cas9 gene-editing system since two years ago. The mutants were finally obtained and the ripening phenotype was observed earlier this month. We found that ripening of fruit from slalkbh2 mutants is delayed, which is similar to that observed in the VIGS experiment. The results and methods are now described in page 17-19 and page 38-39 (highlighted), respectively, in the revised manuscript. In addition, Fig. 10 has been revised.*

Question 2: There are a number of typos which need correcting "Since cnr" alpha-ketoglutaramate for example. This needs a careful review.

*Response: We thank the reviewer for pointing this out. We have read over the entire manuscript to check for the word usage, grammar, and typos. The following words or phrases have been corrected.*

*Page 4: “yeast” has been changed to “yeasts”.*

*Page 6: “demonstrate” has been changed to “demonstrates”; “mediates” has been changed to “mediate”.*

*Page 11: “wild type” has been changed to “wild-type”.*

*Page 12: “mRNA” has been changed to “mRNAs”.*

*Page 13: “Sine” has been changed to “Since”; “SGN Tomato database” has been changed to “Sol Genomics Network (SGN) tomato database”.*

*Page 13 and Page 61: “α-ketoglutaramate” has been changed to “alpha-ketoglutaramate”.*

*Page 14: “participates” has been changed to “participate”.*

*Page 15: “exhibits” has been changed to “exhibited”.*

*Page 20: “mammalian” has been changed to “mammals”; “display” has been changed to “displays”.*

*Page 21: “exhibit” has been changed to “exhibited”.*

*Page 22: “includes” has been changed to “include”; “levels” has been changed to “level”.*

*Page 24: “relative” has been changed to “relatively”.*

*Page 26: “was” has been changed to “were”.*

*Page 29: “was” has been changed to “were”.*

*Page 30: “was” has been changed to “were”.*

*Page 31: “were” has been changed to “was”.*

*Page 35: “were” has been changed to “was”.*

*Page 62: “coexpressed” has been changed to “co-expressed”.*

**Second round of review**

**Reviewer 1**

The authors have made much efforts to address the questions. I still have two questions related with methods before acceptance.

1.	I did not agree the response/answer for Question 4. I have carefully read the literature Duan et al 2017 the authors provided, Figure 2B of Duan et al was the results of LC-MS/MS. These are common knowledge from analytic chemistry. HPLC results showed the A260 values. As known that m6A and A have the same absorbance, the height or area of m6A/A from HPLC results indicate the m6A/A in the sequence. However, the MS signals of m6A and A are different.

2.	m6A-IP-qPCR method: What is the exact meaning of “m6A enrichment”used here ? How does the authors select input mRNA amount as 100 ng? It is hard to understand the calculation equation for m6A enrichment. Whether the amount mRNA used for IP and input led to artificial effects? Since the calculation equation for m6A enrichment is first time used by the authors, they should use spike in RNA with known m6A methylation fraction to demonstrate the accuracy of the analysis. Additionally, the authors did not used any internal RNA as reference gene to control qPCR loading.

**Reviewer 2**

I am fully satisfied by the Response to the Points I raised in my earlier review

**Authors Response**

**Point-by-point responses to the reviewers’ comments:**

Response to the comments of Reviewer #1:

Question 1: I did not agree the response/answer for Question 4. I have carefully read the literature Duan et al 2017 the authors provided, Figure 2B of Duan et al was the results of LC-MS/MS. These are common knowledge from analytic chemistry. HPLC results showed the A260 values. As known that m6A and A have the same absorbance, the height or area of m6A/A from HPLC results indicate the m6A/A in the sequence. However, the MS signals of m6A and A are different.

*Response: We thank the reviewer for this comment. To accurately quantify A and m6A, we have generated standard curves of pure A and m6A by using HPLC. We found that the ratio of peak area of A to equimolar m6A is 0.734, but not 1, suggesting that the ratio of peak area could not accurately indicate the ratio of nucleotide amount. This might be caused by the different molecular weight or molecular structure between m6A and A. Our results are similar to previous study, where the detected amount of A is around 8-fold of m6A by HPLC, whereas the ratio of A to m6A in the sequence used (a mixture of two oligonucleotides in an equimolar ratio) is 12:1 (Mauer et al., 2017. Nature, 541: 371-375; shown in Fig. 1c and Supplementary table 4).*

*In our study, no m6A signal was detected in the presence of SlALKBH2, indicating that SlALKBH2 is indeed an active m6A RNA demethylase.*

Question 2: m6A-IP-qPCR method: What is the exact meaning of “m6A enrichment” used here? How does the authors select input mRNA amount as 100 ng? It is hard to understand the calculation equation for m6A enrichment. Whether the amount mRNA used for IP and input led to artificial effects? Since the calculation equation for m6A enrichment is first time used by the authors, they should use spike in RNA with known m6A methylation fraction to demonstrate the accuracy of the analysis. Additionally, the authors did not used any internal RNA as reference gene to control qPCR loading.

*Response: We thank the reviewer for pointing this out. To avoid confusion, we have rewritten the m6A-IP-qPCR method (page 28-29). The calculation equation for m6A enrichment has been deleted and m6A enrichment is described by “m6A enrichment in specific gene regions was determined by using the cycle threshold (CT) 2(−ΔCT) method [81]. The value for immunoprecipitated sample was normalized against that for ACTIN (Solyc03g078400), which did not show any obvious mRNA m6A peak from m6A-seq data, as an internal control, and then normalized against that for the input.”. According to your suggestion, we have used ACTIN, which did not show any obvious mRNA m6A peak from m6A-seq data, as an internal control to control qPCR loading. The figures (Fig. 2e, 5b, 9d, 10d and Supplementary Fig. S4b) have been revised.*

Finally, we sincerely appreciate you and the reviewers for taking the time to evaluate our manuscript. We are looking forward to hearing from you.
